# Electrochemical synthesis of propylene from carbon dioxide on copper nanocrystals

**DOI:** 10.1038/s41557-023-01163-8

**Published:** 2023-04-06

**Authors:** Jing Gao, Alimohammad Bahmanpour, Oliver Kröcher, Shaik M. Zakeeruddin, Dan Ren, Michael Grätzel

**Affiliations:** 1 Laboratory of Photonics and Interfaces, École Polytechnique Fédérale de Lausanne, Lausanne, Switzerland; 2 Group of Catalysis for Biofuels, École Polytechnique Fédérale de Lausanne, Lausanne, Switzerland; 3 Paul Scherrer Institute, Villigen, Switzerland; 4 School of Chemical Engineering and Technology, Xi’an Jiaotong University, Xi’an, China

**Keywords:** Electrocatalysis, Sustainability

## Abstract

The conversion of carbon dioxide to value-added products using renewable electricity would potentially help to address current climate concerns. The electrochemical reduction of carbon dioxide to propylene, a critical feedstock, requires multiple C–C coupling steps with the transfer of 18 electrons per propylene molecule, and hence is kinetically sluggish. Here we present the electrosynthesis of propylene from carbon dioxide on copper nanocrystals with a peak geometric current density of −5.5 mA cm^−2^. The metallic copper nanocrystals formed from CuCl precursor present preponderant Cu(100) and Cu(111) facets, likely to favour the adsorption of key *C_1_ and *C_2_ intermediates. Strikingly, the production rate of propylene drops substantially when carbon monoxide is used as the reactant. From the electrochemical reduction of isotope-labelled carbon dioxide mixed with carbon monoxide, we infer that the key step for propylene formation is probably the coupling between adsorbed/molecular carbon dioxide or carboxyl with the *C_2_ intermediates that are involved in the ethylene pathway.

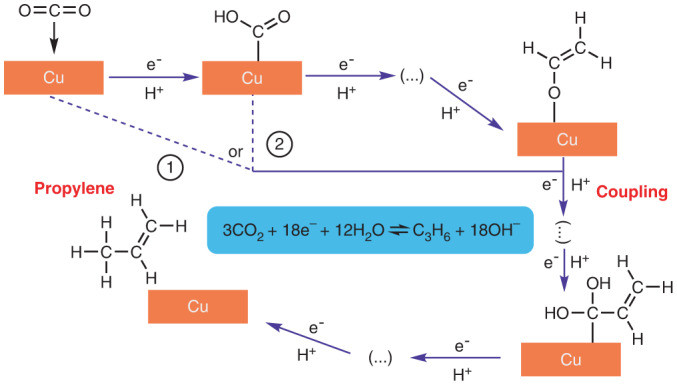

## Abstract (web summary)

The electrochemical reduction of carbon dioxide to propylene requires multiple C–C coupling steps and the transfer of 18 electrons, posing kinetic and thermodynamic barriers. Now the electrosynthesis of propylene from carbon dioxide using copper nanocrystals has been demonstrated, with analysis revealing that the key step for its formation is probably the coupling between carbon dioxide or carboxyl with intermediates involved in the ethylene pathway.

## Main

The electrochemical conversion of carbon dioxide (CO_2_) to value-added products driven by renewable electricity serves as a promising strategy for alleviating the negative impact of excessive anthropogenic carbon emissions^1,2^. With copper-based catalysts, electrochemical CO_2_ reduction has shown an appreciable activity for the production of multiple C_1_ (refs. ^3–6^) and C_2_ chemicals^7–9^. Although C_3+_ terminal oxygenates such as *n*-propanol and *n*-butanol could be produced from CO_2_ reduction^10–12^, C_3+_ hydrocarbons, such as propylene (CH_2_=CH–CH_3_), have rarely been observed as products. Propylene, a critical chemical feedstock, has reached an annual global capacity of 130 Mt in 2019, requiring an input of energy equivalent to the one from about 190 million barrels of crude oil and entailing around 80 Mt of CO_2_ emission (https://cen.acs.org/energy/Periodic-Graphics-Environmental-impact-industrial/97/i24). The electrosynthesis of propylene from CO_2_, yielding a negative carbon footprint, is an attractive strategy for producing this indispensable feedstock for the polymer industry and is yet to be achieved.

The electroreduction of CO_2_ to propylene involves the transfer of 18 electrons per propylene molecule and requires multiple C–C coupling steps^13^, posing kinetic and thermodynamic barriers for driving this reaction:

$$3{\mathrm{CO}}_2 + 12{\mathrm{H}}_2{\mathrm{O}}+18{\mathrm{e}}^-\rightleftarrows{\mathrm{C}}_3{\mathrm{H}}_6+18{\mathrm{OH}}^-\enspace E^{\circ}=0.13\,{\mathrm{V}}\,{\mathrm{versus}}\,{\mathrm{RHE}}$$

where *E*° is the thermodynamic equilibrium potential and RHE represents the reversible hydrogen electrode. All potentials cited in this work are scaled against RHE unless otherwise stated.

Lee et al. observed propylene formation from electrochemical CO_2_ reduction over chloride-induced biphasic Cu_2_O–Cu catalysts with a low production rate of 72 μA cm^−2^ and a Faradaic efficiency (FE) of 0.9% at −1.8 V versus RHE^14^. Recently, Pablo-García et al. proposed that the production of propylene could be traced to the allyl alkoxy (CH_2_=CHCH_2_O) intermediate, easy desorption of which in an alkaline microenvironment results in the unfavourable production of propylene^15^. This conclusion helps to explain why propylene is rarely being produced/detected in CO_2_ reduction, which contrasts with the production of ethylene^16–18^. In-depth understanding of the reaction pathway towards propylene formation is warranted for designing catalysts for this reaction.

Here we synthesize copper nanocrystals (CuNCs), the surfaces of which predominantly consist of Cu(100) and Cu(111) facets, enabling the electrosynthesis of propylene from CO_2_ reduction with appreciable selectivity and production rate. By carrying out well-designed control experiments, including the reductions of CO, CO_2_/CO, CO_2_/He and ^13^CO_2_/CO mixtures, we propose that propylene generation shares a highly protonated *C_2_ intermediate with ethylene generation, and *CO is unlikely to be the *C_1_ intermediate that couples with *C_2_ species for propylene formation. This contrasts with the *n*-propanol pathway where *CO is proposed to be the key *C_1_ precursor participating in the *C_1_–*C_2_ coupling.

## Results

### Characterization of electrocatalyst

**Fig. 1:**
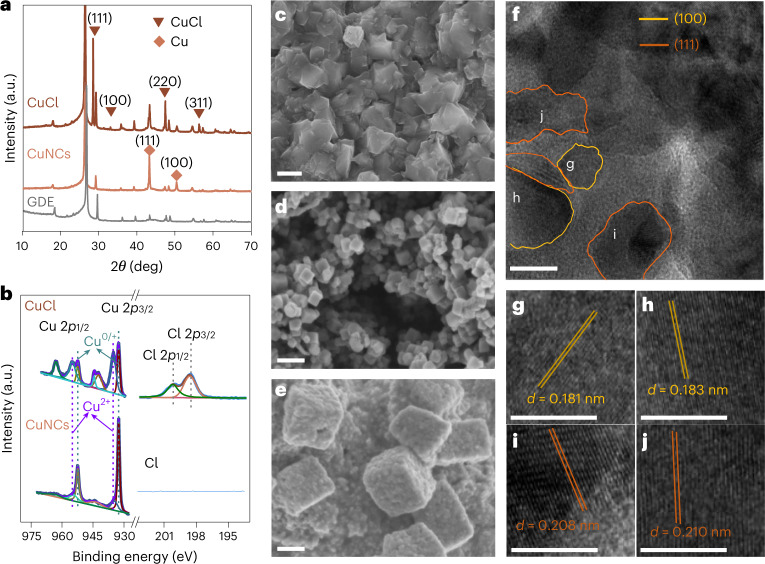
Structural and chemical characterizations of Cu CuNCs. **a**, X-ray diffractograms of CuCl film, pre-reduced CuNCs and GDL substrate. *θ* denotes the angle of X-ray incidence. **b**, High-resolution XPS spectra of Cu 2*p* and Cl 2*p* of CuCl and pre-reduced CuNCs. **c**–**e**, Scanning electron micrographs of CuCl film formed by roughening of sputtered copper film (**c**), CuNCs after pre-reduction (**d**) and CuNCs after CO_2_ reduction at −0.60 V versus RHE for 10 min (**e**). **f**, High-resolution transmission electron micrographs of CuNC catalysts. **g–j**, Lattice fringes of Cu(100) facet (**g**,**h**) and Cu(111) facet (**i**,**j**). *d* in (**g**–**j**) denotes the interplanar spacing of each facet. Scale bars: **c**, 500 nm; **d**,**e**, 100 nm; **f,** 10 nm; **g**–**j**, 5 nm. Source data

**Extended Data Fig. 1:**
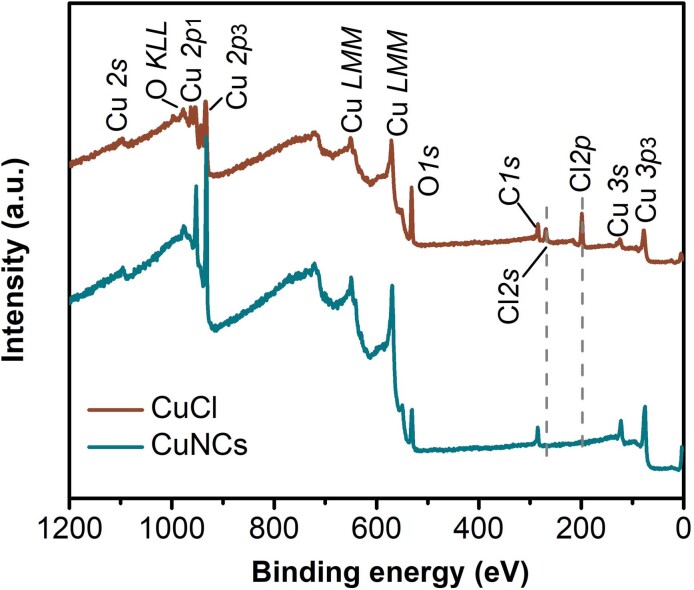
X-ray photoelectron spectra. Survey spectra of as-prepared CuCl layer and CuCl-derived Cu nanocrystals after the pre-reduction at a constant current density of −30 mA cm^−−2^ for ~80 s. The peaks that are assigned to Cl *2p* and Cl *2* *s* disappear after the pre-reduction of CuCl film in an aqueous electrolyte containing KOH and CsI. Source data

A CuCl layer, resulting from an electrochemical roughening of a copper film on a gas diffusion layer (GDL, Supplementary Fig. 1)^19^, was electrochemically pre-reduced to form copper NCs. X-ray diffraction and high-resolution X-ray photoelectron spectroscopy (XPS) confirm the presence of CuCl after electrochemical roughening (Fig. 1a,b and Extended Data Fig. 1)^20^. The surface Cu^2+^ species shown by XPS may result from the oxidation of Cu^0^/Cu^+^ once the sample is exposed to air and moisture (Fig. 1b). CuCl exhibits aggregated cuboids ∼500 nm in size (Fig. 1c). After pre-reduction in the CsI-containing KOH electrolyte, the film shows metallic copper features with dominant Cu(100) and Cu(111) facets (Fig. 1a) and the disappearance of chloride from the XPS spectrum demonstrates the effective removal of residual chloride via pre-reduction (Fig. 1b and Extended Data Fig. 1). The surface after pre-reduction shows densely arranged copper nanoparticles and nanocubes 30–80 nm in size (Fig. 1d). We note here that CsI has little effect on forming nanocubes during pre-reduction (Supplementary Fig. 2). However, keeping electrolytes for pre-reduction and subsequent CO_2_ reduction identical is essential for avoiding any possible reconstruction of copper, indirectly caused by air exposure during electrolyte replacement (Supplementary Fig. 2).

CuNCs were then used as electrocatalysts and characterized after 10 min of CO_2_ reduction at −0.60 V versus RHE. The surface undergoes a further reconstruction and CuNCs agglomerate into rough cubic particles of 100–200 nm (Fig. 1e), consistent with previous reports^19,21^. The representative high-resolution transmission electron micrograph shows lattice fringes of 0.181–0.183 nm and 0.208–0.210 nm (Fig. 1f), representing the presence of Cu(100) and Cu(111) facets (Fig. 1g–j)^22^, respectively. The boundaries where Cu(100) and Cu(111) facets conjoined are also discernible.

### Electrosynthesis of propylene from CO_2_ on CuNC catalyst

**Extended Data Fig. 2:**
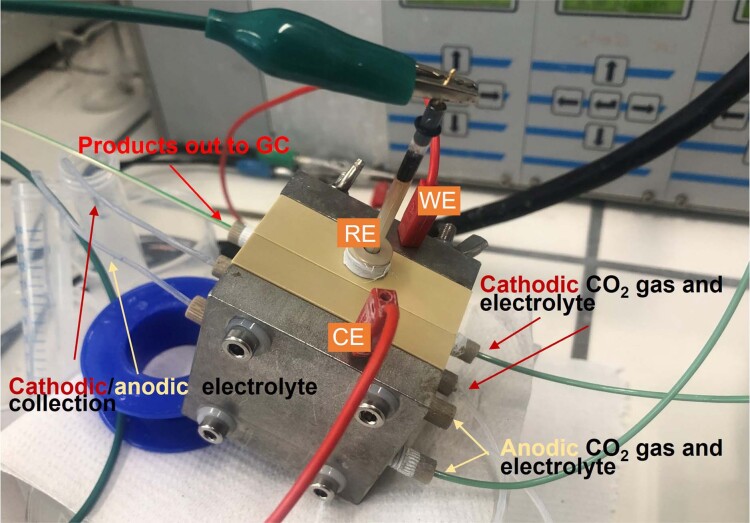
A photograph of the custom-built electrochemical flow cell. The flow cell is divided into four plates which are used for flowing cathodic gas, cathodic electrolyte, anodic electrolyte and anodic gas, respectively. The generated gaseous products in CO_2_ matrix were automatically injected into an online gas chromatography every ~16 min. The liquid products dissolved in catholyte were collected during electrolysis. Here anolyte was also collected and used to quantify the small amount of formate and acetate that crossed over from catholyte via anion exchange membrane.

In an electrochemical flow cell (Extended Data Fig. 2), CuNCs catalyse the conversion of CO_2_ with high reaction rates at moderate potentials in aqueous 1 M KOH with additional 0.2 M CsI (refs. ^23,24^) (Supplementary Fig. 3). For example, the total geometric current density reaches about −0.6 A cm^−2^ with a partial current density towards CO_2_ reduction of about −0.4 A cm^−2^ at −0.675 V.

**Fig. 2:**
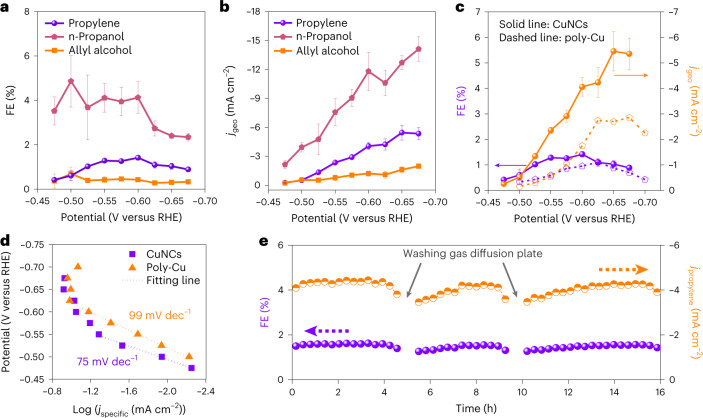
Production of C_3_ products on CuNC catalysts during CO_2_ reduction in an electrochemical flow cell. **a**,**b**, FE (**a**) and partial current density (**b**) of allyl alcohol, propylene and *n*-propanol on CuNCs as a function of applied potential. *j*_geo_ denotes the current density normalized against the geometric area of the catalysts. **c**, FE (purple line) and partial current density (orange line) of propylene on CuNCs (solid line) and polycrystalline copper (dashed line). **d**, Tafel analysis for propylene production on CuNC and polycrystalline copper catalysts. **e**, FE (purple line) and partial current density (orange line) of propylene production over 16 h of electrolysis on CuNCs. Each data point for CuNCs in **a**–**c** corresponds to the average value of three independent measurements from freshly prepared samples and the error bar represents the standard deviation of these measurements. Each data point for polycrystalline copper in **c** corresponds to the average value of two independent measurements from freshly prepared samples. Source data

**Extended Data Fig. 3:**
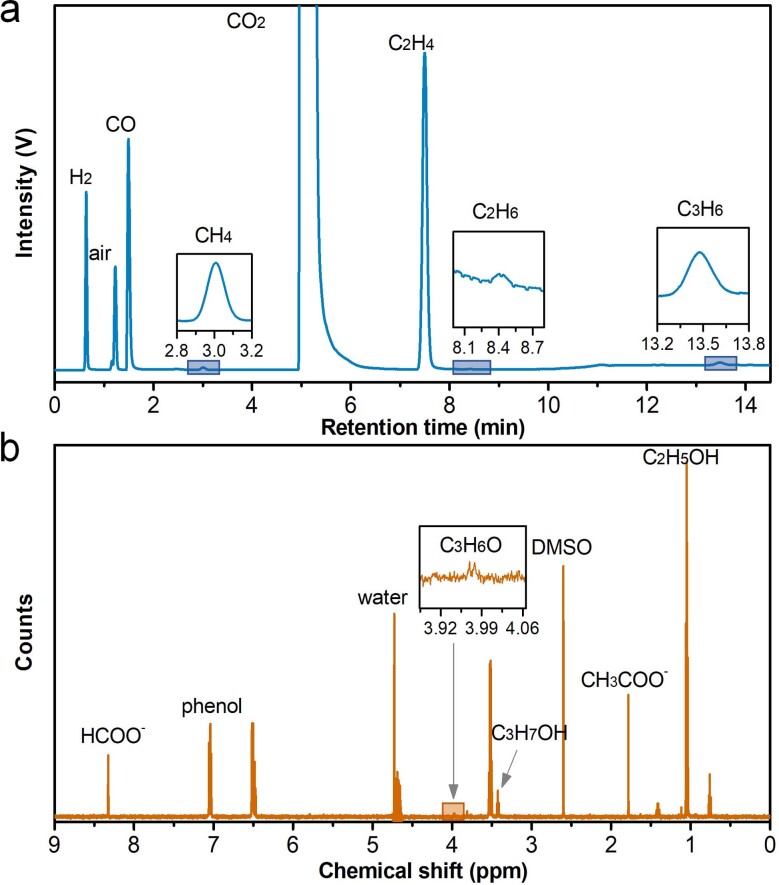
Representative gas chromatograph and ^1^H nuclear magnetic resonance spectrum. Representative (a) Gas chromatograph and (b) ^1^H NMR spectrum recorded in CO_2_ reduction on CuNCs. The area of the liquid product peaks on the right of water peak was normalized against the area of DMSO peak at 2.6 ppm, and the area of the product peaks on the left of water peak is normalized against the area of phenol peak at 7.2 ppm. Source data

The products detected on our CuNC catalyst include C_1_–C_3_ hydrocarbons/oxygenates and hydrogen (Extended Data Fig. 3 and Supplementary Tables 1 and 2). Strikingly, the formation of propylene emerges at a potential of only −0.475 V (Fig. 2a), corresponding to an overpotential of ∼600 mV. If a more negative bias is applied, the FE of propylene increases and reaches a maximum value of 1.4% at −0.60 V, which is 1.6-fold higher than the one reported by Lee et al. on Cu/Cu_2_O biphasic catalysts in a H-type cell^14^. A cathodic current density of 5.46 mA cm^−2^ for propylene production is achieved at −0.65 V (Fig. 2b), delivering an improvement factor of 65 as compared with the previously reported value^14^. The formation of C_3_ chemicals, including propylene, *n*-propanol and allyl alcohol, is observed on CuNCs over a potential range from −0.475 to −0.675 V, with the highest total FE of 6.2% at −0.50 V (Fig. 2a) and the maximum cathodic current density of 21.4 mA cm^−2^ at −0.675 V (Fig. 2b). As a comparison, sputtered polycrystalline copper films that consist of 50–80 nm particles deliver a poorer performance towards propylene formation with an optimized FE of 1.1% peaking at −0.625 V and a partial current density of −2.89 mA cm^−2^ at −0.675 V (Fig. 2c, Supplementary Fig. 4 and Tables 3 and 4).

The surface active sites of CuNC and polycrystalline copper catalysts were further assessed by lead underpotential deposition (UPD) and hydroxide (OH^−^) adsorption to reveal the difference in their catalytic activity. Two cathodic peaks at approximately −0.02 and −0.06 V versus RHE, which could be respectively assigned to the UPD of lead on Cu(111) and Cu(100) facets, are observed (Supplementary Fig. 5)^25–27^. The area ratios of Cu(111) and Cu(100) were calculated to be 1.3 for CuNCs and 2.7 for polycrystalline copper, respectively (Supplementary Table 5). On the other hand, OH^−^ adsorption peaks on Cu(100) and Cu(111) are present at ~0.36 and ~0.46 V versus RHE^28,29^, respectively (Supplementary Fig. 6). The area ratios of Cu(111):Cu(100) determined from OH^−^ adsorption for both CuNC and polycrystalline copper catalysts are consistent with the ones calculated from the lead UPD (Supplementary Table 6). Moreover, OH^−^ adsorption on both catalysts after 10 min of electrolysis at −0.60 V shows that the distribution of facet orientations remains almost unchanged (Supplementary Fig. 7 and Supplementary Table 6). Note that noticeable background currents were observed in the above cyclic voltammograms due to the unavoidable oxygen and carbon component within the porous GDL substrate. Switching the substrate to a non-porous one, such as glass, renders a flat baseline and shows consistency with the above analysis (Supplementary Fig. 8 and Supplementary Table 5). Nevertheless, we selected to perform cyclic voltammetry with the GDL as the substrate to assess the real active sites in the flow cell.

We then normalized the partial current of propylene against the electrochemical surface area of two catalysts (Supplementary Table 5). Interestingly, CuNCs rival polycrystalline copper in terms of the intrinsic activity towards propylene formation, as evidenced by *j*_specific_ and turnover frequency (TOF) (see calculation in Methods and Supplementary Figs. 9 and 10). The kinetics based on the Tafel analysis of propylene also reveal that CuNCs outperform polycrystalline copper with a smaller Tafel slope of 75 mV dec^−1^ (Fig. 2d). The enhanced intrinsic activity of CuNCs may be due to their in situ reconstruction, resulting in an appropriate distribution of Cu(100) and Cu(111) facets on a rough surface, as indicated by the lead UPD and OH^−^ adsorption measurements (Supplementary Figs. 8 and 9). Previous studies have proposed that a mixture of different copper facets is active for catalysing CO_2_ conversion to multi-carbon products^25,28^. The propagation of carbon chains is thermodynamically and kinetically favoured on Cu(100)^30,31^, while coexistence of Cu(111) could provide a conjoined interface for stabilizing key intermediates for multi-carbon products^22^.

**Extended Data Fig. 4:**
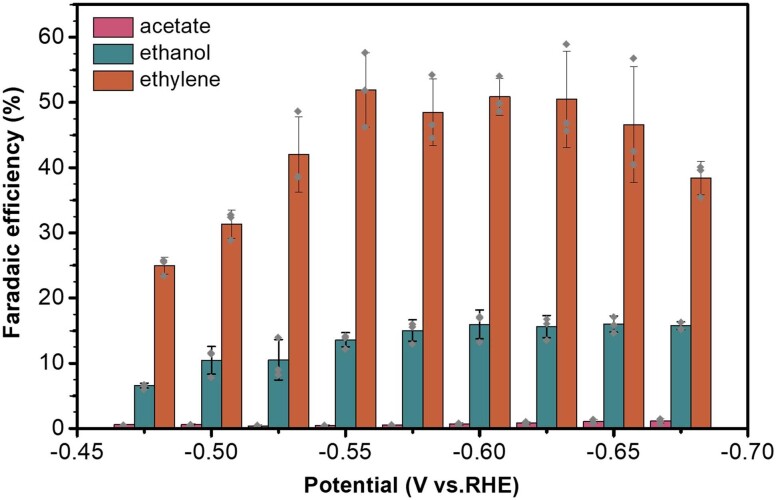
Faradaic efficiency of other products. Faradaic efficiency of acetate, ethanol and ethylene produced on CuNCs catalysts in CO_2_ reduction under different applied potentials. Each data point corresponds to the average of three independent chronopotentiometric measurements obtained from freshly prepared samples and the error bars represent the standard deviations of these measurements. Source data

Besides C_3_ products, C_2_ hydrocarbons and oxygenates are also produced with remarkable selectivity and reaction rate. The FE of C_2_ products increases from 32.1% to 66.0% once the applied potential shifts from −0.475 to −0.550 V and remains at around 60% from −0.550 V to −0.675 V (Extended Data Fig. 4). Meanwhile, the FE of methane is suppressed to <0.4% regardless of the applied potential (Supplementary Fig. 11). At −0.55 V, the FE ratio between C_2+_/CH_4_ reaches a value of up to 1,200, showing the excellent selectivity of CuNCs in catalysing C–C coupling. The partial current density for C_2_ products peaks at −335.5 mA cm^−2^ (Supplementary Fig. 12).

The addition of Cs^+^, although it does not induce any notable morphological difference in the CuNCs (Supplementary Fig. 13), improves both the activity of CO_2_ reduction and hydrogen evolution, regardless of the anion component of the additives (Supplementary Fig. 14). First, larger metal cations such as Cs^+^ with a softer hydration shell have a higher concentration near the surface of the catalyst and deliver a favourable coordination with negatively charged intermediates, that is, CO_2_^−^, thus promoting the CO_2_ reduction rate^32^. Second, hydration of Cs^+^ ions could induce a lower local pH, which improves the activity of CO_2_ reduction by dissolving more CO_2_ molecules, and also promotes the kinetics of hydrogen evolution^33,34^. Moreover, change in the local electric field introduced by hydrated Cs^+^ may also help improve both the CO_2_ reduction rate and hydrogen evolution rate^24,35^. The complexity of the Cs^+^ effects leads to the observed trend: with the addition of 0.2 M CsOH into 1 M KOH electrolyte, selectivity of C_2+_ products is enhanced at potentials greater than −0.6 V versus RHE and hydrogen selectivity is improved at all the tested potentials (Supplementary Fig. 14). It is also noted that the catalyst requires 100 mV smaller overpotential to achieve the optimum formation of C_2+_ products in the presence of Cs^+^, consistent with our recent finding that current density is a critical factor determining C–C coupling activity^36^. On the other hand, the I^−^ anions also could improve the geometric current density at potentials less than −0.60 V if we compared the activity measured in CsOH + KOH electrolyte and CsI + KOH (Supplementary Fig. 15)^37^. Ogura et al. proposed that the specifically adsorbed halides facilitate the electron flow from the electrode surface to the vacant orbital of CO_2_ (ref. ^38^). Akhade et al. reported that a small quantity of KI improves current density at more negative potentials on a copper electrode by enhancing the reaction energetics of *CO coupling due to the presence of I^−^ ions in the electrochemical double layer^39^. This is consistent with the increased CO_2_ reduction current observed on our CuNCs at potentials less than −0.6 V (Supplementary Fig. 15). We also observed that the addition of I^−^ suppresses methane formation, different from the results shown in the study by Strasser and co-workers in which an enhanced methane formation was reported^40^. This difference is probably due to the higher local pH in our alkaline reaction system that facilitates the generation of hydrocarbon product.

In summary, the distribution of cationic and anionic species in the local microenvironment could induce complex impacts, such as stabilization of intermediate, specific adsorption on electrode and repelsion of reacting species, which could impair or favour CO_2_ reduction. In our system, the presence of Cs^+^ and I^−^ in the electrolyte was found to increase the CO_2_ electroreduction rate on CuNC catalysts without sacrificing their intrinsic selectivity towards C_2+_ products at potentials greater than −0.60 V versus RHE.

**Extended Data Fig. 5:**
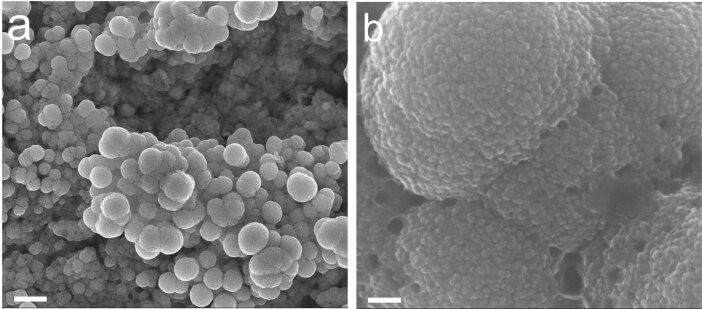
Scanning electron micrographs of Cu nanocrystals after 16-hr reduction. The reduction was performed at −0.60 V versus RHE (−273.7 mA cm^−2^). Scale bars: 1 μm for a and 100 nm for b. Source data

The stability of our CuNCs was evaluated by 16 h electrolysis at −273.7 mA cm^−2^, corresponding to a potential of −0.60 V versus RHE (Fig. 2e and Supplementary Fig. 16). With the periodic removal of precipitated salt (Supplementary Fig. 17)^41,42^, CuNCs show durable performance towards propylene formation with the FE and partial current density remaining at 95% of the initial values after 16 h (Fig. 2e). However, we observed a reconstruction of the catalyst from nanocubes to nanospheres that consist of agglomerates with particle sizes of 10–30 nm (Extended Data Fig. 5). This could be due to the high surface free energy of nanocubes which induces aggregation after long-term electrolysis^43^, although the possibility of this morphology change originating from an unavoidable oxidation during cell disassemby could not be ruled out. We further performed OH^−^ adsorption on the CuNC catalyst immediately after 16 h CO_2_ reduction (Supplementary Fig. 18). The OH^−^ adsorption peaks on both Cu(100) and Cu(111) facets are still present at ~0.37 and ~0.48 V versus RHE, respectively. Further analysis of their charges evidenced a ratio of 1.05 between the active area of Cu(111) and Cu(100) (Supplementary Table 7), implying that the feature of nearly equal distribution of Cu(100) and Cu(111) facets remains almost unchanged despite the huge morphology reconstruction.

### Identification of the intermediates for propylene production

**Fig. 3:**
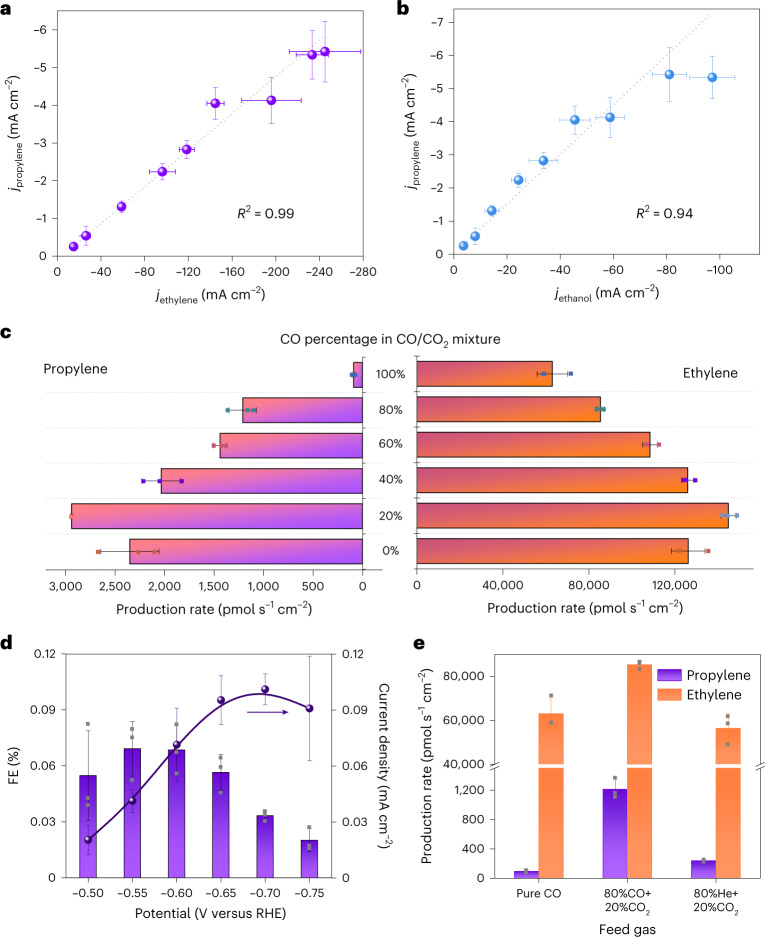
Identification of the key intermediates for propylene production. **a**,**b**, Partial current density of propylene as a function of partial current density of ethylene (**a**) and ethanol (**b**). **c**, Production rate of propylene and ethylene during CO/CO_2_ co-feeding experiments with different CO percentages in the CO/CO_2_ mixture. **d**, FE and partial current density of propylene detected from CO reduction at different potentials. **e**, Production rate of propylene and ethylene over CuNC catalyst at −0.60 V versus RHE under three different feeding conditions. Each data point in **c**–**e** corresponds to the average of chronopotentiometric measurements obtained from three independent and freshly prepared samples and the error bar represents the standard deviation of these measurements. Source data

**Extended Data Fig. 6:**
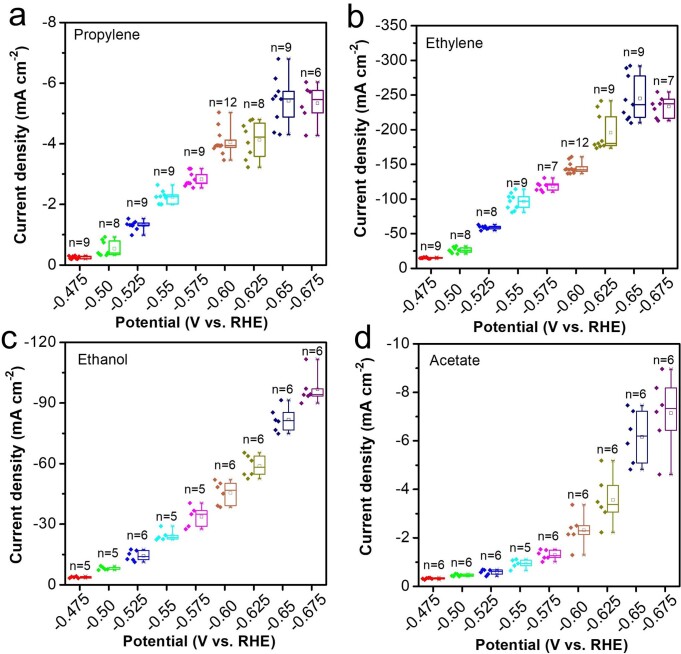
Statistical distribution of the partial current densities of different products. The distribution of partial current densities of (a) propylene, (b) ethylene, (c) ethanol and (d) acetate in CO_2_ reduction at different potentials. Data were analysed from multiple independent experiments and the total number n of the data points at each potential has been indicated in the figures. The minimum, maximum and average value of each group data have been denoted as − , − and □. The box is bounded by the upper (75%) and lower (25%) quartiles and the median (50%) is drawn as a line in the box. The range of whisker is determined by the minima and maxima points of each group data. Source data

The substantial production rate of propylene achieved on our CuNCs makes this material an excellent model catalyst for further mechanistic analysis^15^. With the general belief that the formation of C_3_ compounds involves a key step of coupling between *C_1_ and *C_2_ species^44^, we first elucidate the structure of *C_2_ species by analysing the linear correlation between the partial current density (*j*) of propylene and the *j* of a specific C_2_ product (a statistical analysis is shown in Extended Data Fig. 6). The linearity between *j*_propylene_ and *j*_C2_, as indicated by the *R*^2^ values of the fitting curves, becomes poorer if the C_2_ product contains more oxygen atoms (${{R}}_{\mathrm{propylene - ethylene}}^2 > {{R}}_{\mathrm{propylene - ethanol}}^2 > {{R}}_{\mathrm{propylene - acetate}}^2$; Fig. 3a,b and Supplementary Fig. 19). This indicates that *C_2_ intermediates involved in *C_1_–*C_2_ coupling for propylene production are probably highly protonated ones, such as *OCH=CH_2_ (ref. ^45^). We also introduced CO into the feed gas to form mixtures of CO/CO_2_ to tune the production rate of the products of interest (Fig. 3c–e and Supplementary Tables 8–10)^46,47^. Strikingly, the absolute production rate of propylene also follows the same trend as that of ethylene (Fig. 3c). For example, with 20% of CO_2_ being substituted by CO, the production rate of ethylene is enhanced by 14% compared with the one using 100% CO_2_. Simultaneously, the production rate of propylene increases by 25%.

**Extended Data Fig. 7:**
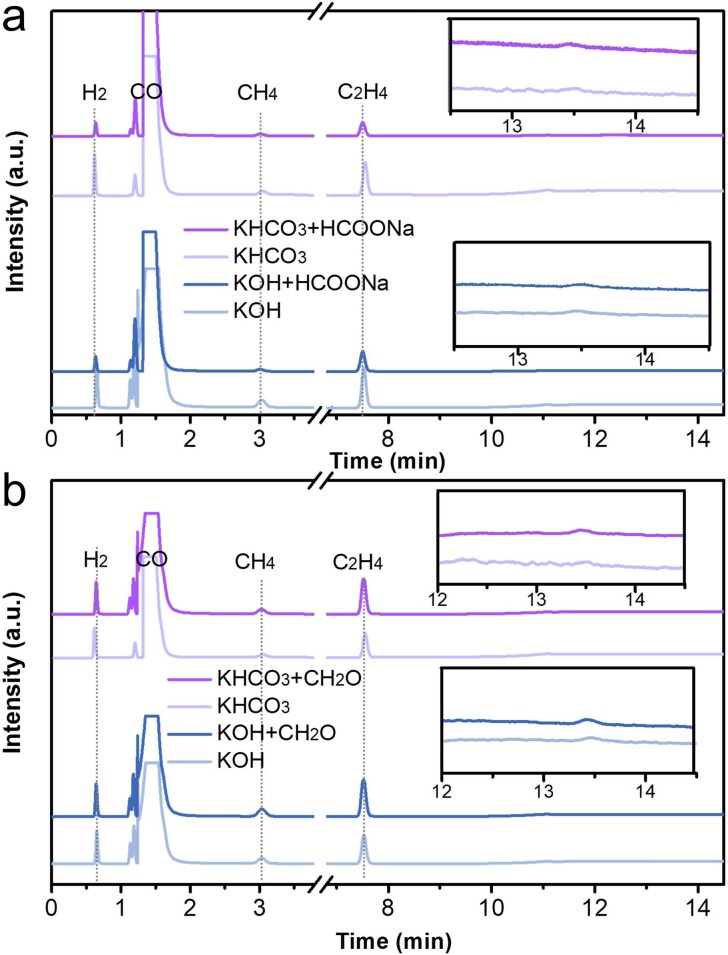
Gas chromatographs collected from the reduction of carbon monoxide. The reduction was performed at −0.60 V versus RHE in 1 M KOH or 0.5 M KHCO_3_ containing (a) 50 mM HCOONa and (b) 10 mM CH_2_O. Source data

Surprisingly, only a trace amount of propylene is detected from the reduction of 100% CO with a cathodic partial current density of <0.10 mA cm^−2^ and a production rate of <90 pmol s^−1^ cm^−2^ at potentials from −0.50 to −0.75 V versus RHE (Supplementary Tables 9 and 10) although CuNCs are still capable of producing sufficient *C_2_ intermediates as indicated by the FE of ethylene (Fig. 3d and Supplementary Figs. 20 and 21). This interesting observation highlights that the active *C_1_ intermediates for *C_1_–*C_2_ coupling towards propylene formation might be missing in CO reduction. First, although formate is not a product of CO reduction^45^, the possibility of *OCO^−^ as the *C_1_ intermediate is disproved by co-reduction of CO and HCOO^−^ which shows a low rate of <35 pmol s^−1^ cm^−2^ for propylene production, similar to the value observed in CO reduction (Extended Data Fig. 7). Secondly, all *C_1_ intermediates involved in the pathway of CO → CH_4_ conversion are unlikely to be involved in *C_1_–*C_2_ coupling for propylene production because the reduction of CO leads to the appreciable formation of methane (Supplementary Table 9). Moreover, the possibility of formaldehyde being the key *C_1_ intermediate, as proposed by a recent mechanistic study on the routes towards C_3_ products^15^, is also excluded by carrying out CO reduction using formaldehyde-containing electrolytes (Extended Data Fig. 7). Thus, the key *C_1_ fragments involved in propylene pathway are likely to be two species, that is, molecular/adsorbed CO_2_ or *COOH. This is strongly corroborated by the observation that the production rate of propylene increases by a stunning factor of 14, from 86 pmol s^−1^ cm^−2^ in CO reduction to 1.24 × 10^3^ pmol s^−1^ cm^−2^ in the reduction of a mixture comprising 80% CO and 20% CO_2_ (Fig. 3e). Note that the reduction of an 80% He + 20% CO_2_ mixture leads to a production rate of 200 pmol s^−1^ cm^−2^, corresponding to only 1/6 of the value observed in the reduction of 80% CO + 20% CO_2_ mixtures (Fig. 3e). Hence, *C_1_ intermediates resulting from CO_2_ or reduction of *CO_2_ couple with the *C_2_ intermediates stemming primarily from CO reduction, leading to the production of propylene with substantially improved rate in the reduction of 80% CO + 20% CO_2_.

In contrast, the pathway towards the formation of *n*-propanol is slightly different. It is proposed that *CO is the key *C_1_ species for *n*-propanol production^10,22,48^. On the basis of our observation that the maximum production of *n*-propanol occurs if large amounts of CO and C_2_H_4_ are simultaneously formed (Supplementary Tables 1 and 3), we propose that the coupling of *CH_2_CH/*CH_3_CH and *CHO(H)/*CO leads to the formation of *n*-propanol. This is consistent with the finding of Pablo-García et al., showing that the lowest activation barrier for C_3_ backbone formation is the coupling of CH_2_CH + CHO or CH_3_CH + CHO(H), where CHO(H) is formed from *CO hydrogenation^15^. The difference in the structure of *C_1_ intermediate between the propylene pathway and the *n*-propanol pathway leads to the formation of *n*-propanol being less affected by the change of feed gas (Supplementary Table 8) because the *CO intermediate could be either due to the direct adsorption of feed CO or from the reduction of CO_2_. Because the local pH becomes higher and the number of protons decreases with the incorporation of CO into the reactant stream due to the fact that CO does not react with OH^−^ like CO_2_, the production rate of *n*-propanol declines slightly once the percentage of CO increases in the CO/CO_2_ mixture (Supplementary Fig. 22). For allyl alcohol, the change of production rate with different reactant mixtures is difficult to ascertain due to the low levels of allyl alcohol produced, approaching the detection limit of ^1^H NMR (Supplementary Fig. 22).

### Quantitative gas chromatography–mass spectrometry analysis for ^13^CO_2_/^12^CO reduction

**Fig. 4:**
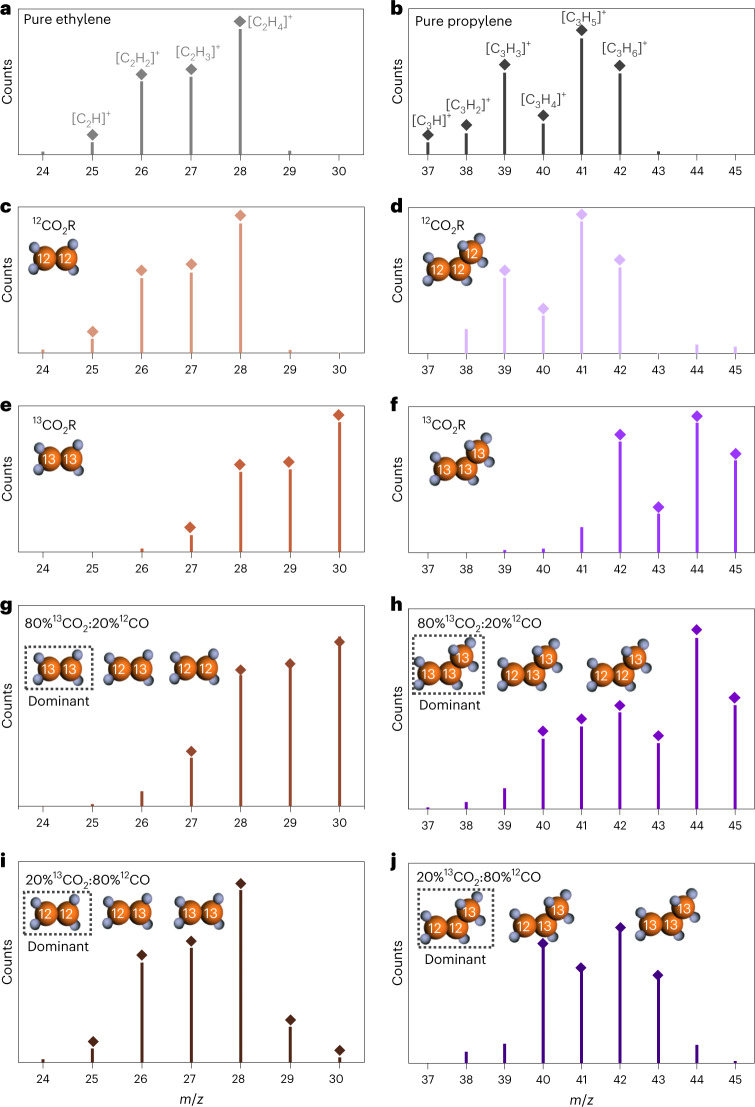
GC–MS analysis for the reduction under different feeding conditions. **a**,**b**, Mass spectra of standard gas of ethylene (**a**) and propylene (**b**). **c**,**e**,**g**,**i**, Mass spectra of ethylene under ^12^CO_2_ feeding (**c**), ^13^CO_2_R feeding (**e**), 80% ^13^CO_2_ and 20% ^12^CO co-feeding (**g**) and 20% ^13^CO_2_ and 80% ^12^CO co-feeding (**i**) conditions. **d**,**f**,**h**,**j**, Mass spectra of propylene under ^12^CO_2_ feeding (d), ^13^CO_2_ feeding (**f**), 80% ^13^CO_2_ and 20% ^12^CO co-feeding (**h**) and 20% ^13^CO_2_ and 80% ^12^CO co-feeding (**j**) conditions. Source data

We further combined isotopic labelling experiments with gas chromatography–mass spectrometry (GC–MS) to gain more insights into the *C_2_ and *C_1_ intermediates for C–C coupling to propylene production. Standard gas of ethylene and propylene show consistent mass-to-charge signals (*m/z*) compared to the standard mass spectra of two chemicals (Fig. 4a,b and Supplementary Fig. 23).

Ethylene and propylene produced from ^12^CO_2_ reduction show the same ionized molecules and fragments as compared to their respective standard gas (Fig. 4c,d), with the two highest peaks at *m/z* = 28 and *m/z* = 41 representing ^12^C_2_H_4_^+^ and ^12^C_3_H_5_^+^, respectively. In the reduction of ^13^CO_2_, the *m/z* ratios of these two highest peaks increase by 2 (*m/z* = 30, ^13^C_2_H_4_^+^) and 3 (*m/z* = 44, ^13^C_3_H_5_^+^) for ethylene and propylene, respectively (Fig. 4e,f), indicating the production of ^13^C_2_H_4_ and ^13^C_3_H_6_.

If the ratio of ^13^CO_2_/^12^CO is 80%/20%, the highest peak of ethylene appears at *m/z* = 30, representing the dominant presence of ^13^C_2_H_4_. The peaks at *m/z* = 29 and 28 exhibit slightly higher intensity compared to the standard ^13^C_2_H_4_ spectrum (Fig. 4g), indicating the additional formation of ^13^CH_2_^12^CH_2_ (from the ^13^CO_2_–^12^CO pathway) and ^12^C_2_H_4_ (from the ^12^CO–^12^CO pathway), with a low percentage of ^12^C_2_H_4_. The MS signals of propylene also show a wide range of *m/z* ratio from 40 to 45, with the main fragment peaks locating at 44 and 45 (Fig. 4h), indicating the formation of ^13^C_3_H_6_.

The mass spectrum of ethylene detected from the reduction of the mixture of ^13^CO_2_/^12^CO = 20%/80% shows the highest peak at *m/z* = 28 and two smaller peaks at *m/z* = 29 and 30 (Fig. 4i), indicating that the majority of the formed ethylene has two ^12^C atoms. In comparison, the mass spectrum of propylene exhibits the strongest peak at *m/z* = 42, which is similar to the reference ^12^C_3_H_6_ (Fig. 4d) except that the *m/z* value is shifted by 1 unit (Fig. 4j). Additional weak peaks located at *m/z* = 44 and 45 are also observed. This result indicates that the majority of propylene has two ^12^C atoms and one ^13^C atom and arises from the coupling of ^13^CO_2_/*^13^COOH intermediates with the *^12^C_2_ species that are produced from ^12^CO reduction. This quantitative analysis supports our hypothesis that the key *C_1_ and *C_2_ intermediates for propylene generation are likely to be molecular/adsorbed CO_2_ or *COOH and highly protonated *C_2_, that is, *OCH=CH_2_, respectively.

### Identification of *C_3_ intermediates for propylene generation

**Fig. 5:**
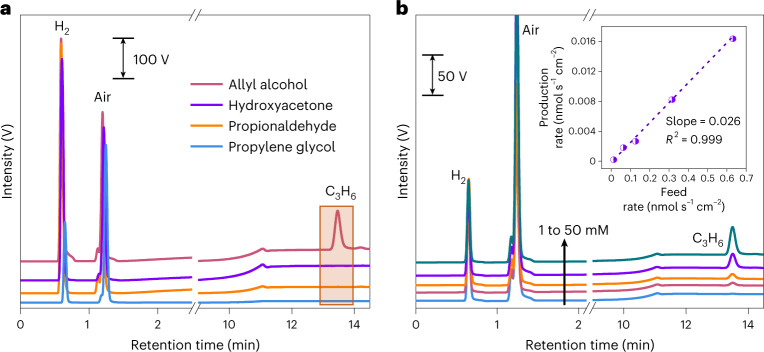
Identification of *C_3_ intermediates for propylene generation. **a**, Online gas chromatographs collected during electrochemical reduction of possible C_3_ intermediates. **b**, Online gas chromatographs collected during electrochemical reduction of allyl alcohol with different concentrations of 1, 5, 10, 25 and 50 mM. The vertical black arrow indicates that the concentration increases from 1 mM to 50 mM sequentially. Inset: production rate of propylene as a function of the feed rate of allyl alcohol. Source data

To gain further insights into the key *C_3_ intermediates for propylene production, we performed the electrochemical reduction of allyl alcohol, propionaldehyde, hydroxyacetone and propylene glycol by dissolving them in KOH + CsI electrolyte. The possibility of decomposition of these compounds in alkaline solution over the test period is ruled out by the observation of their fingerprint signals in ^1^H NMR spectra after electrolysis. Interestingly, only the reduction of allyl alcohol leads to the production of propylene, and propylene is absent without applied bias (Fig. 5a and Supplementary Fig. 24), consistent with a recent study showing a noticeable yield of propylene from electroreduction of allyl alcohol on oxide-derived copper catalyst^15^. We also reveal a linear relationship between the generation rate of propylene and the feed amount of allyl alcohol (Fig. 5b), but only <3.0% of propylene produced from CO_2_ reduction could originate from the direct reduction of allyl alcohol precursor (see calculation in Methods and Supplementary Tables 11 and 12), indicating that the major *C_3_ intermediate for propylene production is unlikely to be allyl alcohol.

## Discussion

**Fig. 6:**
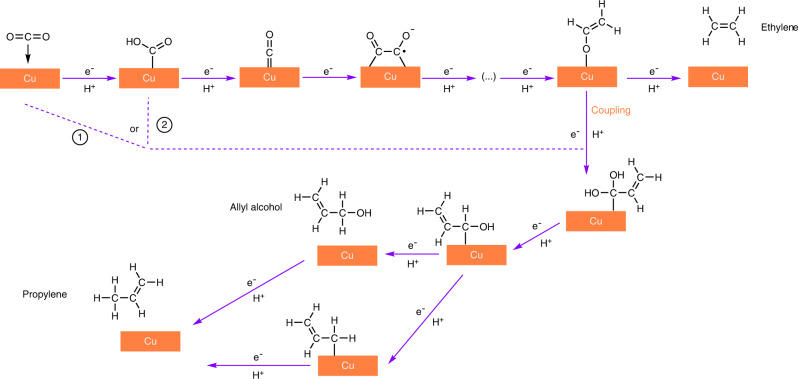
Proposed mechanism for the electroreduction of CO_2_ to propylene on copper catalyst. CO_2_ is first reduced to *CO intermediate. The coupling between two *C_1_ intermediates leads to the formation of *C_2_ intermediate, which is further reduced to C_2_ products such as ethylene. The coupling between possible *C_1_ and *C_2_ intermediates leads to the formation of C_3_ products such as propylene. The direct reduction of allyl alcohol also leads to the formation of propylene.

Based on the above mechanistic analysis, we highlight the key steps and crucial intermediates for propylene production (Fig. 6). CO_2_ is first reduced to *COOH, which undergoes further reduction to *CO with elimination of a water molecule^45^. These *CO intermediates could undergo C–C coupling to form *C_2_ species, which are hydrogenated to form either ethylene or ethanol^45,49,50^, with the former being favoured on our CuNC catalyst. Some of the adsorbed *C_2_ intermediates that feature a carbon double bond and are highly protonated, such as *OCH=CH_2_, could undergo coupling with either molecular/adsorbed CO_2_ or *COOH intermediates, followed by multiple proton-coupled electron transfer steps to form allyl alcohol and propylene.

**Extended Data Fig. 8:**
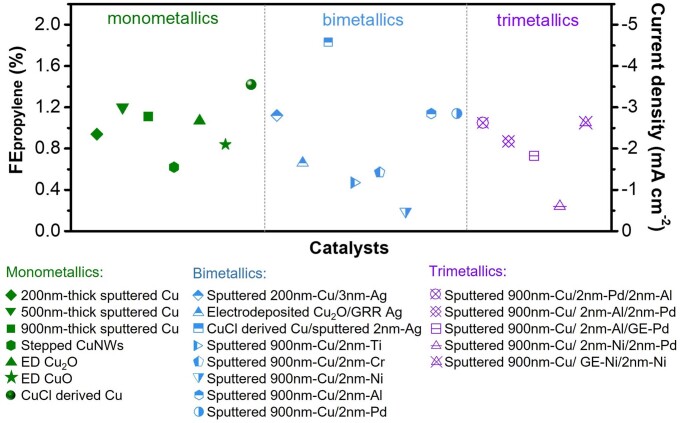
Faradaic efficiency and partial current density of propylene on various catalysts. All the reduction reactions on Cu based monometallics, bimetallics and trimetallics were performed at a cathodic current density of 250 mA cm^−2^ and the electrolyte used here was 1 M KOH. Source data

The production of propylene on our CuNCs benefits from their nanostructure. The catalyst is composed of nanocrystals with prevalent Cu(100) and Cu(111) facets, providing active centres for binding the key *C_1_ and *C_2_ species and improving the intrinsic activity of CuNCs towards propylene production as compared to polycrystalline copper. The CuNCs are endowed with specific sites for CO_2_ reduction, allowing them to reach substantial geometric current densities for propylene formation. We further investigated a large library of copper-based monometallic, bimetallic and even trimetallic electrocatalysts for improved propylene generation (Extended Data Fig. 8). We have achieved a peak FE of 1.83% for propylene generation with CuNCs coated with a 2 nm silver layer, which may result from the increased ethylene production due to the improvement of local CO concentration by the introduction of silver active sites. Moreover, enhancing the flow rate of CO_2_ to 20 cm^3^ min^−1^ leads to an increase in FE_propylene_ by 14% on the CuNCs (Supplementary Fig. 25).

Transfer of 18 electrons is required for the production of one propylene molecule from three CO_2_ molecules. The intermediate species involved in this process are more numerous than the ones revealed by our study. Nevertheless, the mechanism uncovered in our study offers opportunities for designing advanced catalysts for the efficient production of this crucial chemical feedstock. Although still far from large-scale practical implementation, this study opens a pathway to the electrosynthesis of propylene via electrochemical CO_2_ reduction.

## Methods

### General

All chemicals are used as received without further purification. Deionized water (18.2 MΩ, Millipore) was used for preparing solutions and washing samples.

### Synthesis of CuCl-derived copper catalysts

The synthesis procedure for the CuCl layer was modified from a previous study^1^. Here, a 920 nm-thick Cu film (99.995%) was deposited via sputtering (DP650, Alliance-Concept) onto a GDL (38BC, Fuel Cell Store). The Cu/GDL substrate was then electrochemically roughened in 0.1 M KCl electrolyte by repeating five cycles of triangular potential scans. Each triangular potential scan includes three steps: (1) a chronoamperometric step at an applied potential of 0.24 V versus RHE for 10 s; (2) a linear sweep voltammetric step from 0.24 to 1.74 V versus RHE at a scan rate of 500 mV s^−1^; (3) a chronoamperometric step at an applied potential of 1.74 V versus RHE for 5 s. The as-prepared GDL-supported CuCl films were then rinsed thoroughly with deionized water and dried using compressed air. The CuCl films were pre-reduced in a custom-designed flow cell at a constant current density of −30 mA cm^−2^ for ~80 s. The aqueous electrolyte was prepared by dissolving 56.11 g of KOH (Reactolab) and 51.96 g of CsI (99.999%, Alfa Aesar) into 1 litre of deionized water.

### Characterizations of catalysts

The surface morphologies of copper-based samples were acquired using a field emission scanning electron microscope (Zeiss Merlin). Image acquisition was carried out via an in-lens detector under an accelerating voltage of 15 kV. High-resolution transmission electron micrographs of the catalysts were obtained using a transmission electron microscope (Talos, FEI) equipped with a high-angle angular dark field detector. X-ray diffraction was measured on a Bragg–Brentano instrument (Cu Kα radiation, *λ* = 1.5409 Å) with a grazing incident beam. XPS was performed using a PHI VersaProbe II scanning XPS microprobe (Physical Instruments). Analysis was carried out using a monochromatic Al Kα X-ray source of 24.8 W power with a beam size of 200 µm.

### Electrochemical CO_2_ reduction and product analysis

The electrolysis was performed using a Gamry (Interface 1000) potentiostat and each reduction reaction lasted for ∼3,000 s. *iR* correction was made automatically via a current interrupt mode. A custom-built flow cell containing a cathodic chamber and an anodic chamber separated by an anion-exchange membrane (Fumasep FKS-50, Fumatech) was used (Supplementary Fig. 3). The catholyte was identical with the one used for pre-reduction (1 M KOH containing 0.2 M CsI). The anolyte was prepared by dissolving 56.11 g of KOH into 1 litre of deionized water. The as-prepared catholyte and anolyte were respectively pumped into the cathodic and anodic liquid chambers at the same flow rate of 0.25 cm^3^ min^−1^. Before and during the electrochemical reaction, the cathodic and anodic gas chambers were flowed continuously with various feeds at rates of 10 and 5 cm^3^ min^−1^, respectively, controlled by two mass flow controllers (Alicat Scientific). The flow rate of the cathodic chamber was also confirmed at the GC outlet by a soap-bubbled volumetric meter. The gases employed in this study include ^12^CO_2_ (99.999%, Carbagas), ^13^CO_2_ (Sigma-Aldrich, 99.0 at% ^13^C), ^12^CO (99.997%, Carbagas), helium (99.9999%, Carbagas) and their mixtures. The ratio between ^12^CO_2_/^12^CO, ^13^CO_2_/^12^CO or ^12^CO_2_/He was tuned by adjusting the corresponding flow rate of each gas, with the total flow rate being 10 cm^3^ min^−1^.

For each measurement, fresh working electrodes and electrolytes were used. The geometric surface area of the working electrode was 0.33 cm^2^. A gas diffusion layer with sputtered 200-nm-thick platinum (99.995%) was used as the counter electrode and a Ag/AgCl electrode (saturated KCl, Pine) was used as the reference electrode. The electrochemical potential of the Ag/AgCl was calibrated daily against an RHE (HydroFlex, Gaskatel) immersed in 0.1 M HClO_4_ solution, prepared by diluting 0.862 ml of HClO_4_ (70%, ACS reagent, Merck) into 100 ml deionized water. All of the potentials measured in this work were referenced to the RHE using the following conversion:

$${{E}}_{{{{\mathrm{RHE}}}}}\left( {{{\mathrm{V}}}} \right) = {{E}}_{{{{\mathrm{Ag}}}}/{{{\mathrm{AgCl}}}}}\left( {{{\mathrm{V}}}} \right) + 0.197 + 0.0591 \times {{{\mathrm{pH}}}}$$

During the electrolysis three gas aliquots were automatically injected into an online GC (Trace ULRTA, Thermo). The first aliquot was sampled at ∼600 s after the start of reaction to ensure adequate equilibrium of gas phase. The gas products were separated by a micropacked shincarbon column (Restek) and quantified by a pulse discharge detector (Vici). The oven was programmed as follows: (1) holding at 60 °C for 3.5 min; (2) increasing to 180 °C with a ramp rate of 40 °C min^−1^ and holding at 180 °C for 2.5 min; (3) increasing to 260 °C with a ramp rate of 40 °C min^−1^ and holding at 260 °C for 3.5 min. The total run time was 14.5 min. The pulse discharge detector signals were calibrated using standard gas mixtures (Carbagas) with all relevant gases, including H_2_, CO, CH_4_, C_2_H_4_, C_2_H_6_ and C_3_H_6_. The liquid products that dissolved in the electrolyte were collected after electrolysis. The electrolyte was mixed with D_2_O (99.9% deuterium, Aldrin) and an internal standard consisting of 25 mM phenol (>99.0%, Sigma-Aldrich) and 5 mM dimethyl sulfoxide (DMSO, 99.7%, Acros Organics). Then the prepared solution was analysed on a ^1^H NMR spectrometer (Avance III HD 600 MHz, Bruker) and water suppression technique was applied (Supplementary Fig. 5). The areas of the product peaks on the left of water peak were normalized against the peak area of DMSO, and the areas of the product peaks on the right of water peak were normalized against the area of phenol. Standard solutions with known concentrations of reference compounds of HCOONa (for HCOO^−^, >99.0%, Fluka Analytical), CH_3_COONa (for CH_3_COO^−^, >99.0%, Sigma-Aldrich), C_2_H_5_OH (≥99.8%, Fisher Scientific), C_3_H_7_OH (≥99.8%, Fisher Scientific) and C_3_H_6_O (>99.0%, Sigma-Aldrich) in 1 M KOH were used for calibration.

The partial current density of each product is calculated by multiplying the FE by the total geometric current density. The average of multiple measurements was used in this work.

### Calculation of TOF

The TOF, in units of nmol s^−1^ cm^−2^, is calculated based on the specific surface areas of catalysts determined by lead UPD measurements:

1 $${\mathrm{TOF}}_{\mathrm{propylene}} = \frac{{j_{\mathrm{propylene}}({\mathrm{specific}})}}{{18 \times 96{,}485.3\,{\mathrm{C}}\,{\mathrm{mol}}^{ - 1}}}$$

where $j_{\mathrm{propylene}}({\mathrm{specific}})$ represents the partial current density of propylene against the specific surface area.

The TOF value given in this work corresponds to the average of three independent measurements.

### Isotope-labelling experiments and product analysis

Isotopically labelled ^13^CO_2_ (Sigma-Aldrich, 99.0 at.% ^13^C) was also used as the reactant gas. Pure ^13^CO_2_ and mixed ^13^CO_2_–^12^CO feeding experiments were carried out using the same protocol as mentioned above. Gas products were collected into a withdrawal valve after the system reached equilibrium and were injected by microsyringe into a GC–MS instrument (Agilent 7890B/5977A). A GC equipped with a capillary column (HP-Plot/Q, Agilent) was used for chemical separation with helium (99.9999%, Carbagas) as the carrier gas. The GC was interfaced with an MS (5977A, Agilent), which was operated with a filament current of 34.6 μA and electron energy of 70 eV in electron ionization mode. The data acquisition and processing were performed with GC/MSC MassHunter Acquisition software. The signals were referenced to National Institutes of Standard (NIST) library.

### Electrochemical OH^−^ adsorption and lead UPD

Electrochemical OH^−^ adsorption on copper active sites was performed by CV using N_2_-saturated 1 M KOH solution with 0.2 M CsI additive as the electrolyte. All the measurements were carried out using the same flow cell as used to perform CO_2_ electroreduction; electrolyte and helium was continuously purged into the corresponding chambers with flow rates of 0.25 cm^3^ min^−1^ and 10 cm^3^ min^−1^, respectively. Both copper-based electrodes were pre-reduced at −30 mA cm^−2^ for ∼80 s and then the potential was kept at 0 V versus RHE for 3 min immediately before measuring OH^−^ adsorption. The CV curves were recorded within a potential range from 0 to 0.6 V versus RHE at a scan rate of 100 or 50 mV s^−1^. The charge of OH^−^ adsorption on the respective facet of Cu(100) and Cu(111) was calculated by integrating the corresponding peak. Reference values of 8.22 and 2.16 μC cm^−2^ were used for calculating the surface areas of Cu(100) and Cu(111), respectively^2^.

Lead UPD measurement was carried out by flowing a N_2_-saturated 0.1 M HClO_4_ solution with 10 mM Pb(OAc)_2_ into the flow reactor. The catalysts were first preconditioned at −30 mA cm^−2^ for ∼80 s and the potential was kept at the initial potential of the CV scans for an additional 3 min, followed by immediately recording the CV curves from −0.12 to 0.31 V versus RHE for CuNCs and from −0.16 to 0.19 V versus RHE for polycrystalline copper at a scan rate of 10 mV s^−1^. The facet areas of Cu(100) and Cu(111) were calculated by normalizing the deposition charge to the reference values: 262 μC cm^−2^ for (100) and 285 μC cm^−2^ for (111), respectively^2^. As a control experiment, the lead UPD of the CuNC and polycrystalline catalysts that were prepared onto the non-porous glass was also conducted in a three-electrode set-up.

### Calculation of the percentage of propylene being produced from the electrochemical reduction of allyl alcohol

The electrochemical reduction of allyl alcohol was carried out in the same flow cell used for CO_2_ reduction. Each reaction was performed at −250 mA cm^−2^ (corresponding to ∼0.60 V versus RHE) for ∼3,000 s and the other parameters were the same as the ones used for CO_2_ reduction. Aqueous 1 M KOH solutions containing 0.2 M CsI additive, with different concentrations of allyl alcohol, that is, 1, 5, 10 and 50 mM, were respectively pumped into the reactor at a flow rate of 0.25 cm^3^ min^−1^. Helium was fed into the cathodic gas camber at a flow rate of 10 cm^3^ min^−1^. The gas products in helium matrix were automatically sampled into an online GC and the collected electrolytes after electrolysis were analysed by ^1^H NMR. The feed rate of allyl alcohol (*v*_f_) and the production rate of propylene (*v*_p_) were first calculated from the following equations:

2 $$v_{\mathrm{f}} = \frac{{c_{\mathrm{AA}} \times V_{\mathrm{electrolyte}}}}{{t_1 \times S_{\mathrm{WE}}}} = \frac{{c_{\mathrm{AA}} \times v_{\mathrm{electrolyte}}}}{{S_{\mathrm{WE}}}}$$

3 $$v_{\mathrm{p}} = \frac{{n_{\mathrm{propylene}}}}{{t_2 \times S_{\mathrm{WE}}}}$$

where *c*_AA_ is the concentration of allyl alcohol;*v*_electrolyte_ is the flow rate of electrolyte (0.25 cm^3^ min^−1^);

*S*_WE_ is the geometric surface area of working electrode (0.33 cm^2^ in our flow cell);

*n*_propylene_ is the number of moles of propylene detected by GC; and

*t*_2_ is the time required to fill up the sample loop (20 μl)

4 $$t_2 = \frac{{V_{\mathrm{gas}}}}{{v_{\mathrm{gas}}}} = \frac{{0.02\,{\mathrm{cm}}^3}}{{10\,{\mathrm{cm}}^3\,{\mathrm{min}}^{ - 1}}} = 0.002\,{\mathrm{min}} = 0.12\,{\mathrm{s}}$$

By plotting *v*_p_ as a function of *v*_f_ at different concentrations of allyl alcohol, a linear correlation between these two parameters was obtained with a slope of 0.026 (Fig. 5b and Supplementary Table 11). Based on this linear correlation and the generated concentration of allyl alcohol detected in CO_2_ reduction, the partial production rate of propylene attributed to the allyl alcohol electroreduction (*v*_p-AAR_) is calculated to be (Supplementary Table 12):

5 $$v_{\mathrm{p} \mbox{-} \mathrm{AAR}} = 0.026 \times \frac{{c_0 \times v_{\mathrm{electrolyte}}}}{{S_{\mathrm{WE}}}}$$

where *c*_0_ represents the concentration of allyl alcohol produced by CO_2_ electrolysis.

The percentage of propylene being produced from the reduction of allyl alcohol (*X*) is calculated as (Supplementary Table 12):

6 $$X = \frac{{v_{\mathrm{p} \mbox{-} \mathrm{AAR}}}}{{v_{\mathrm{p} \mbox{-} \mathrm{total}}}} \times 100{{{\mathrm{\% }}}}$$

where *v*_p-total_ represents the total production rate of propylene

## Online content

Any methods, additional references, Nature Portfolio reporting summaries, source data, extended data, supplementary information, acknowledgements, peer review information; details of author contributions and competing interests; and statements of data and code availability are available at 10.1038/s41557-023-01163-8.

## Supplementary information

Supplementary Information Supplementary Figs. 1–25, Tables 1–12, and refs. 1 and 2.

## Data Availability

The authors declare that all data supporting the results of this study are available within the paper and its Supplementary Information files. Data are also available upon request. Source data are provided with this paper.

## Source data

Source Data Fig. 1 Source data for XRD and XPS, and unprocessed electron micrographs.
Source Data Fig. 2 Statistical source data
Source Data Fig. 3 Statistical source data
Source Data Fig. 4 Statistical source data
Source Data Fig. 5 Statistical source data
Source Data Extended Data Fig. 1 XPS source data
Source Data Extended Data Fig. 3 GC and ^1^H NMR source data
Source Data Extended Data Fig. 4 Statistical source data
Source Data Extended Data Fig. 5 Unprocessed electron micrographs
Source Data Extended Data Fig. 6 Statistical source data
Source Data Extended Data Fig. 7 GC source data
Source Data Extended Data Fig. 8 Source data for faradaic efficiency and partial current density
