# Supplementary material for: Electrochemical synthesis of propylene from carbon dioxide on copper nanocrystals
Source: Nat Chem. 2023 Apr 6;15(5):705–13. doi: 10.1038/s41557-023-01163-8 (PMC10159857; doi:10.1038/s41557-023-01163-8)
Supplement: Supplementary file 1 — Supplementary Figs. 1–25, Tables 1–12, and refs. 1 and 2. [file 41557_2023_1163_MOESM1_ESM.pdf]

nature chemistry

Article https://doi.org/10.1038/s41557-023-01163-8

Electrochemical synthesis of propylene from carbon dioxide on copper nanocrystals

In the format provided by the authors and unedited

Table of contents

1. Supplementary Figures 2

Supplementary characterization of substrate and electrode 2
Supplementary characterization of the CuNCs and poly-Cu catalysts 4
Additional data related to stability tests 17
Supplementary data for identifying the key intermediates for propylene formation 20
Extended experiments for improved propylene formation 26

2. Supplementary Tables 27

3. Supplementary Reference 37

1. Supplementary Figures

Supplementary characterization of substrate and electrode

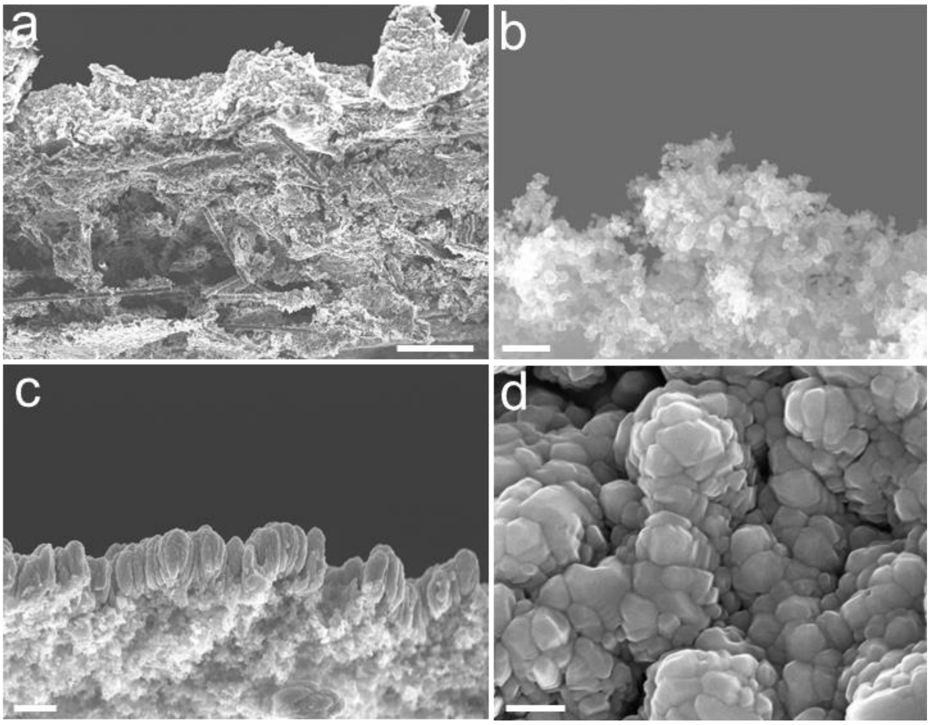

Supplementary Figure 1. Scanning electron micrographs of the substrate. SEM cross-section views of (a) a gas diffusion layer substrate and (b) PTFE particles on top of GDL; (c) SEM cross-section view and (d) surface view of the sputtered poly-Cu particles with an average thickness of ~920 nm and particle size of 100~200 nm. Scale bars: 100 μm for a, 500 nm for b, 1 μm for c and 200 nm for d.

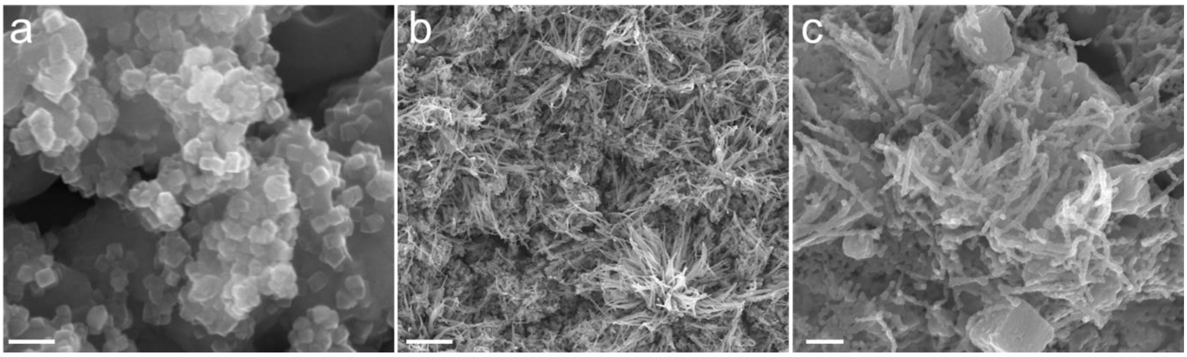

Supplementary Figure 2. Scanning electron micrographs of the catalysts pre-reduced in different electrolytes. (a) SEM image of CuNCs after pre-reduction of CuCl film in 1 M KOH electrolyte at -30 mA cm^{-2} for ~80 s, (b) SEM image and (c) magnified SEM image of the pre-reduced CuNCs after running CO_2 electrolysis at -0.60 V vs. RHE for 10 min in CsI-containing KOH electrolyte. Scale bars: 100 nm for a, 1 μm for b and 200 nm for c. CuNCs after the pre-reduction of CuCl film in KOH electrolyte shows no difference as compared to the one pre-reduced in CsI-containing KOH electrolyte (Figure 1d). Interestingly, SEM images of the Cu NCs after 10 min-CO_2 electrolysis in CsI-containing solution show that observable Cu nanowires that are composed of small nanoparticles distribute over the electrode surface. This might be due to the unexpected oxidation of Cu surface during a short shutdown of electrochemical measurement during replacing the electrolyte[1,2].

Supplementary characterization of the CuNCs and poly-Cu catalysts

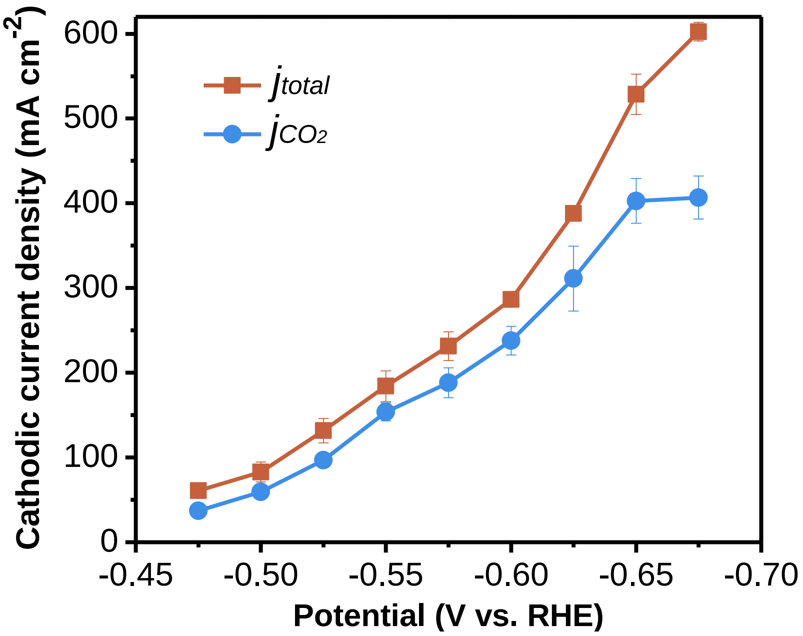

Supplementary Figure 3. Total cathodic current density and partial current density towards CO_2 reduction under different potentials on CuNCs catalysts. The current density is normalized against the geometric surface area. Each data point corresponds to the average of three chronopotentiometric measurements from freshly-prepared samples and the error bar represents the standard deviation of these measurements.

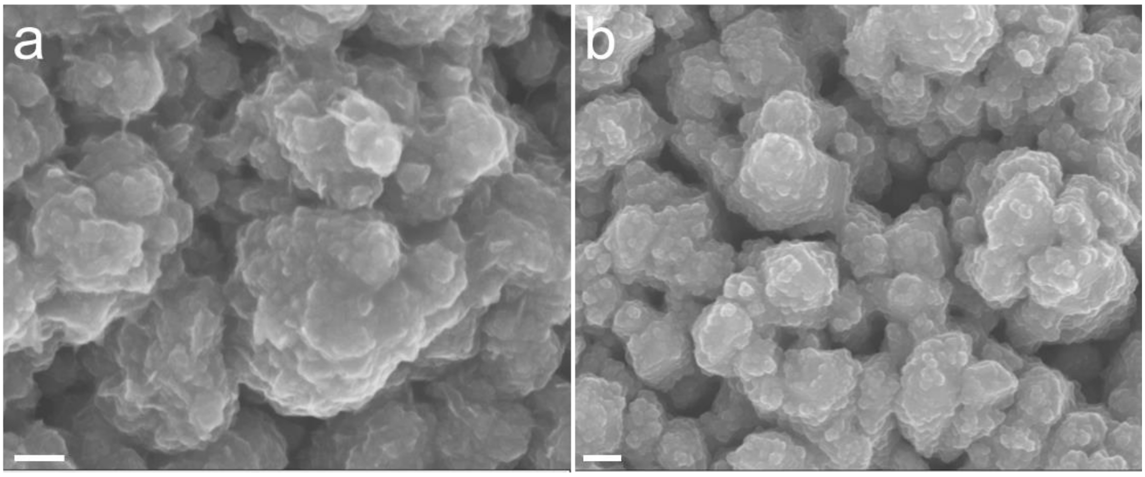

Supplementary Figure 4. Scanning electron micrographs of poly-Cu catalyst. SEM images of sputtered poly-Cu catalyst (a) after pre-reduction at a constant current density of -30 mA cm^{-2} for ~80 s and (b) after 10 min-CO_2 electrolysis at -0.65 V vs. RHE. Scale bars: 100 nm for a and b.

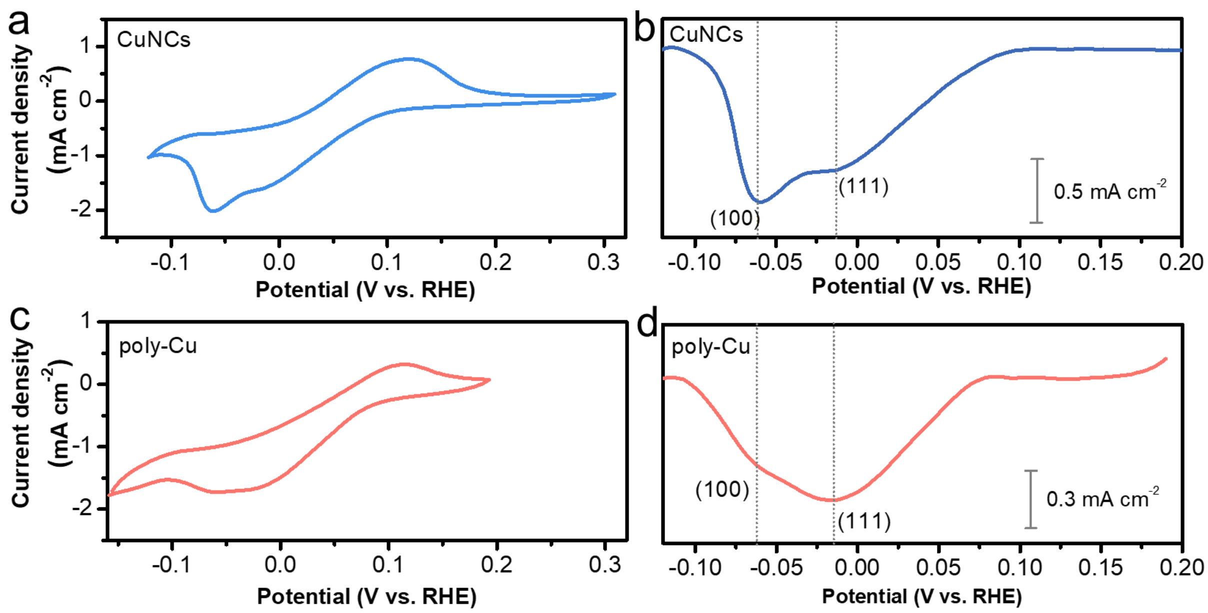

Supplementary Figure 5. Pb underpotential deposition measurements. Cyclic voltammograms recorded on pre-reduced (a-b) CuNCs and (c-d) poly-Cu in 0.1 M $HClO_4$ aqueous solution with 10 mM $Pb(oAc)_2$. (b) and (d) are the zoomed-in view of the cathodic peaks of the cyclic voltammograms, showing the depostion of Pb on Cu(100) and Cu(111) facets. The curves were recorded at a scan rate of 10 mV s^{-1}.

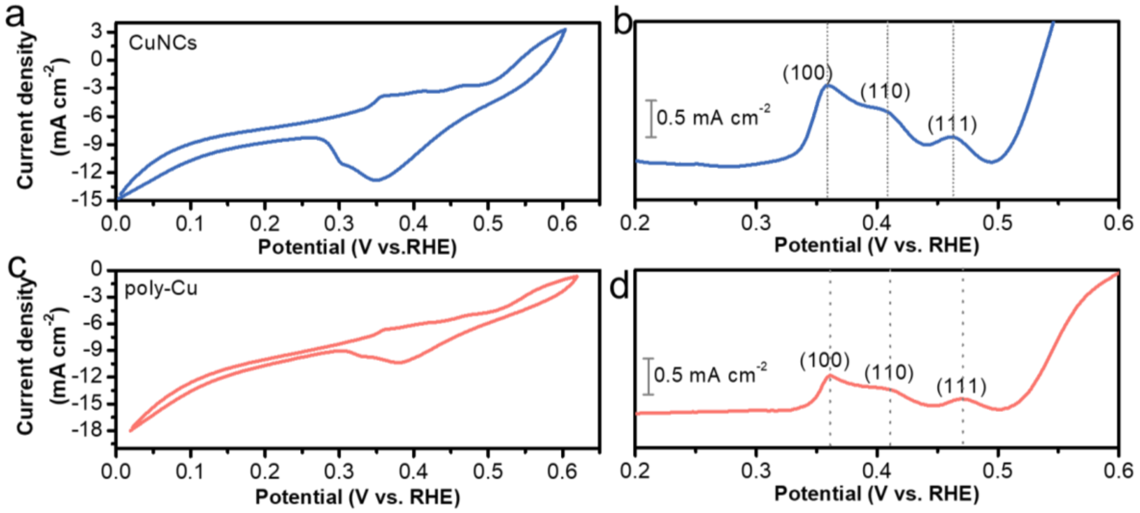

Supplementary Figure 6. OH^- adsorption measurements on pre-reduced catalysts. Cyclic voltammograms recorded on pre-reduced (a-b) CuNCs and (c-d) poly-Cu in 1 M KOH aqueous solution with 0.2 M CsI additive. (b) and (d) are the zoomed-in view of the anodic peaks of the cyclic voltammograms, showing OH^- adsorption peaks on Cu(100), Cu(110) and Cu(111) facets. The curves were recorded at a scan rate of 100 mV s^{-1}.

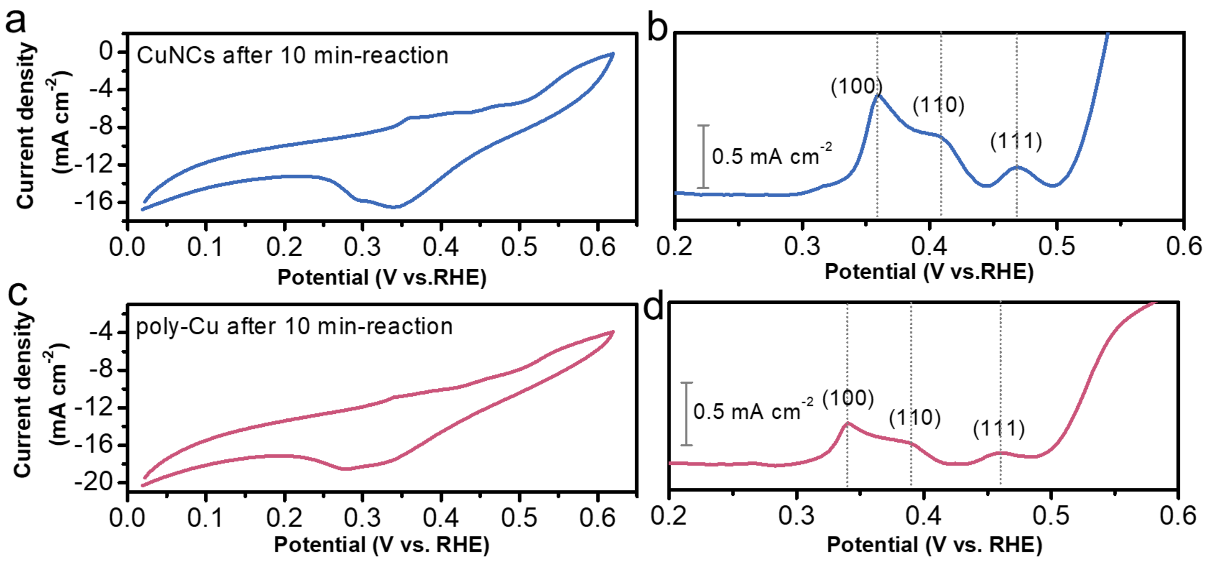

Supplementary Figure 7. Post-electrolysis OH$^-$ adsorption measurements. Cyclic voltammograms recorded on (a-b) CuNCs and (c-d) poly-Cu after running 10 min-CO_2 reduction at -0.60 V vs. RHE. (b) and (d) are the zoomed-in view of the anodic peaks of the voltammograms, showing OH$^-$ adsorption peaks on Cu(100), Cu(110) and Cu(111) facets. The curves were recorded at a scan rate of 100 mV s^{-1}.

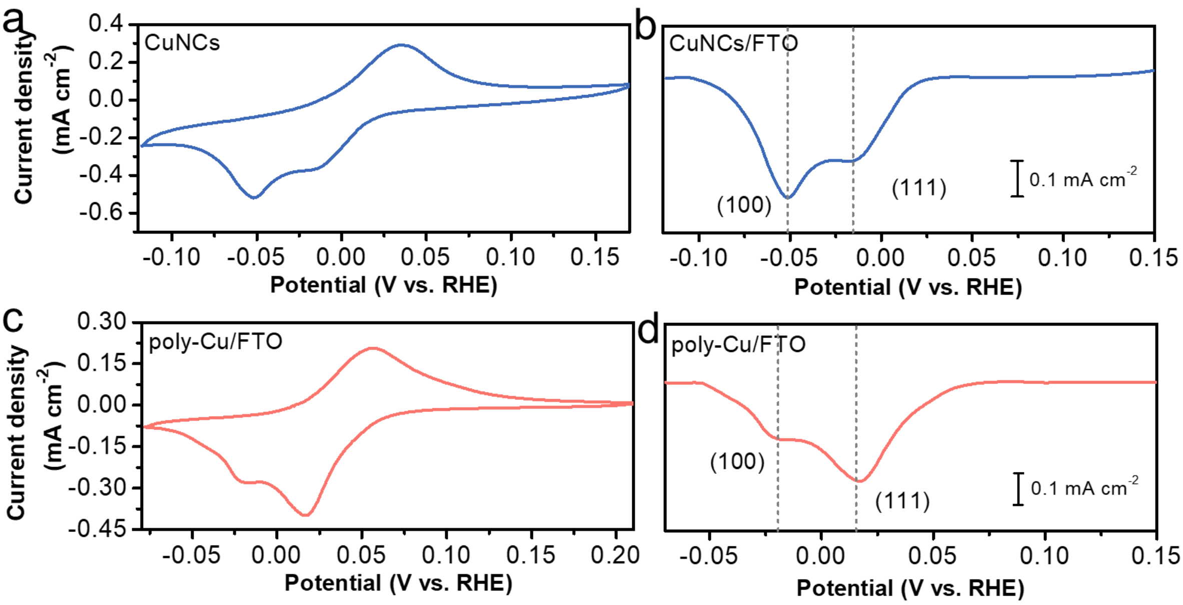

Supplementary Figure 8. Pb underpotential deposition measurements on FTO-supported catalysts. Cyclic voltammograms recorded on pre-reduced (a-b) CuNCs and (c-d) poly-Cu catalysts that were prepared onto non-porous FTO substrate. The solution is 0.1 M $HClO_4$ aqueous solution with 10 mM $Pb(oAc)_2$. (b) and (d) are the zoomed-in view of the cathodic peaks of the cyclic voltammograms, showing the depostion of Pb on Cu(100) and Cu(111) facets. The curves were recorded at a scan rate of 10 mV s^{-1}.

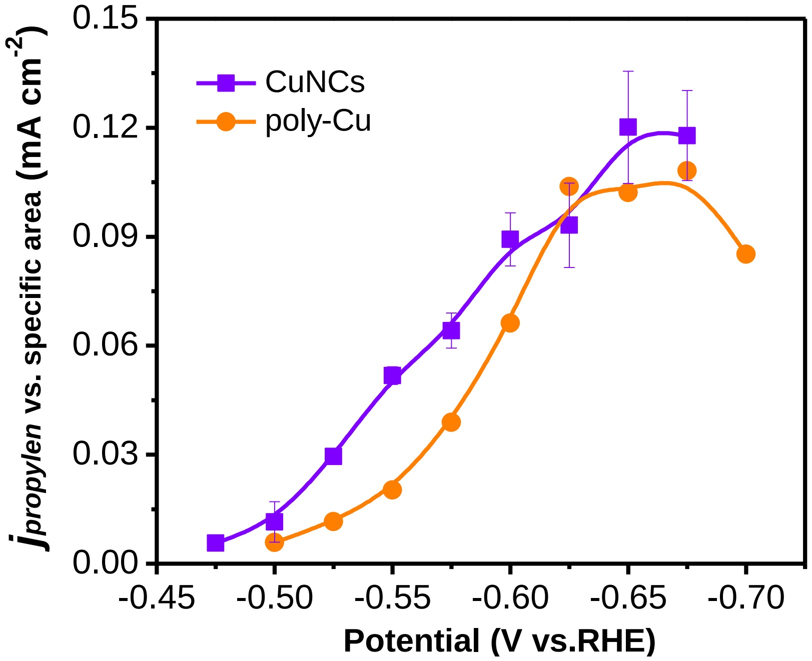

Supplementary Figure 9. Partial current density of propylene normalized against the specific surface area of CuNCs and poly-Cu catalysts determined by Pb UPD measurement. Each data point measured on CuNCs corresponds to the average of three independent chronopotentiometric measurements obtained from freshly-prepared samples and the error bar represents the standard deviation of these measurements. Each data point for poly-Cu corresponds to the average value of two independent measurements from freshly prepared samples.

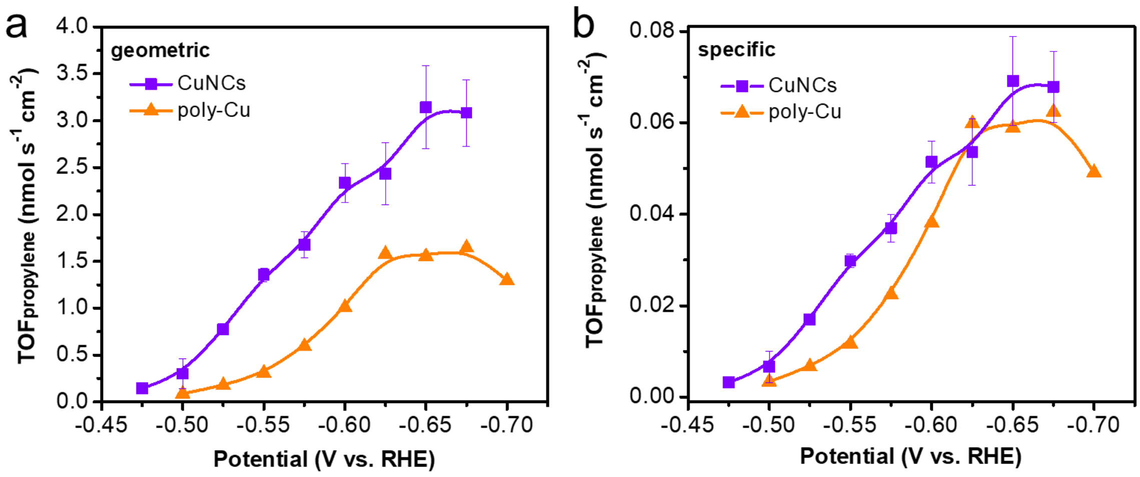

Supplementary Figure 10. Turnover frequency (TOF) of produced propylene. TOF of produced propylene on poly-Cu and CuNCs is normalized against (a) geometric surface area and (b) specific surface area. Each data point measured on CuNCs corresponds to the average of three independent chronopotentiometric measurements obtained from freshly-prepared samples and the error bar represents the standard deviation of these measurements. Each data point for poly-Cu corresponds to the average value of two independent measurements from freshly prepared samples.

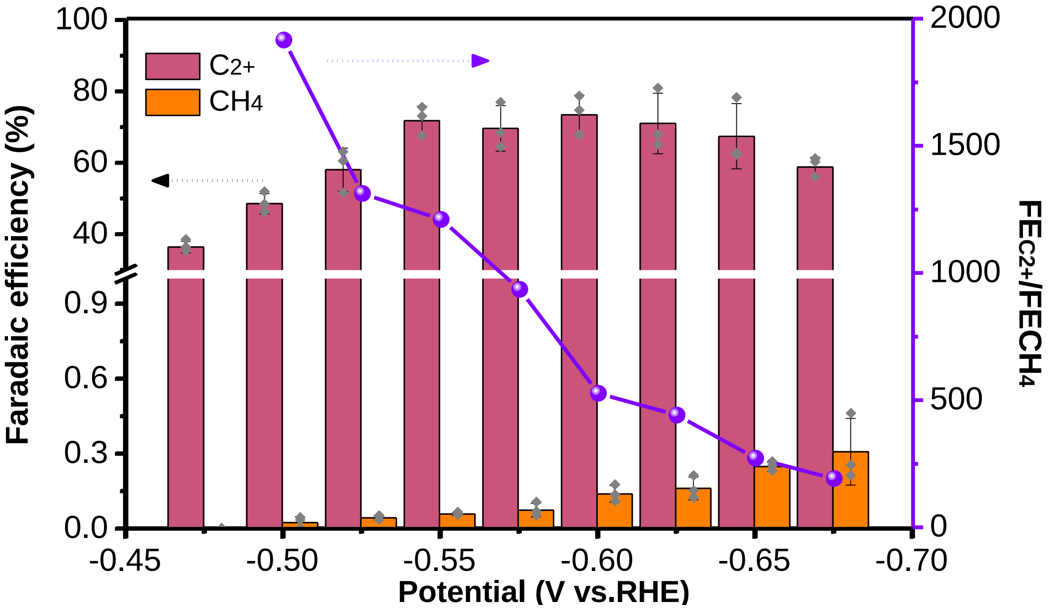

Supplementary Figure 11. Average Faradaic efficiency of C_{2+} products and CH_4, and the Faradaic efficiency ratio between C_{2+} and CH_4 under different applied potential during CO_2 reduction on CuNCs catalyst. Each data point corresponds to the average of three independent chronopotentiometric measurements obtained from freshly-prepared samples and the error bar represents the standard deviation of these measurements.

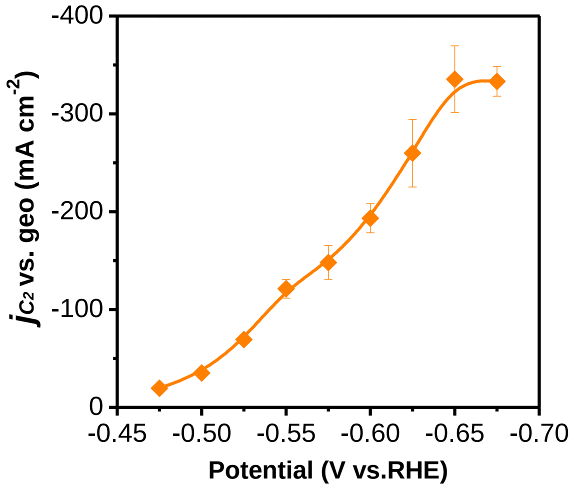

Supplementary Figure 12. Average partial current density of C_2 products produced on CuNCs catalysts during CO_2 reduction in CsI-containing KOH electrolyte. Each data point corresponds to the average of three independent chronopotentiometric measurements obtained from freshly-prepared samples and the error bar represents the standard deviation of these measurements.

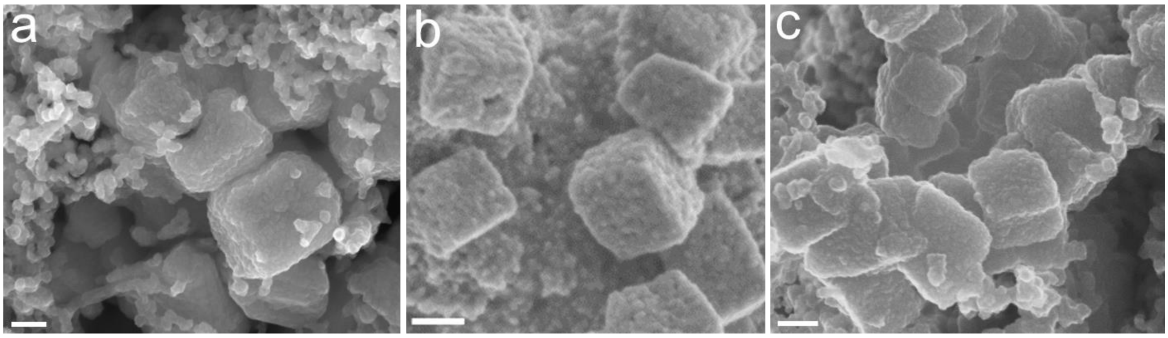

Supplementary Figure 13. Scanning electron micrographs of catalysts prepared in different electrolytes. SEM images of CuNCs after CO_2 reduction at -0.60 V vs. RHE for 10 min in (a) bare KOH, (b) CsI-containing KOH and (c) CsOH-containing KOH electrolytes. Scale bars: 100 nm for a to c.

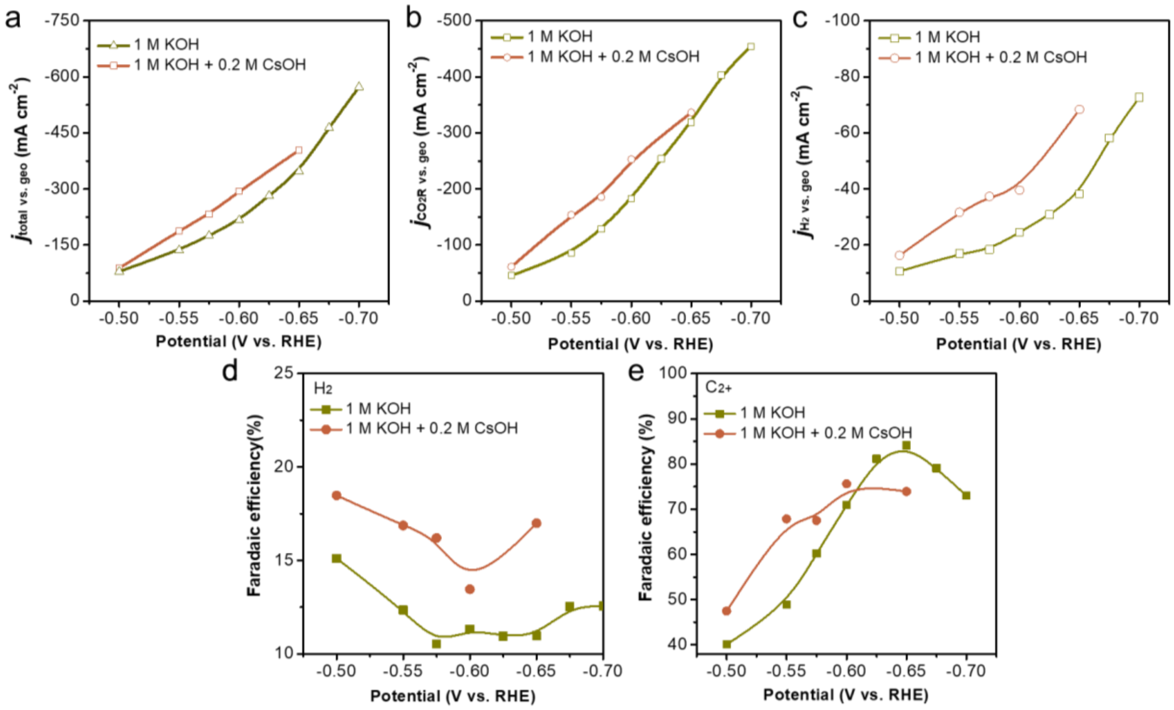

Supplementary Figure 14. Electrochemical performance in Cs^+-containing and Cs^+-free electrolytes. (a) Total current density, (b) partial current density of CO_2 reduction and (c) partial current density of H_2 on CuNCs catalysts in bare KOH electrolyte and KOH containing CsOH; Faradaic efficiency of (d) H_2 and (e) C_{2+} products detected on CuNCs catalysts during CO_2 reduction in different electrolytes. The current density is normalized against geometric surface area. Each data point in (a-e) corresponds to the average value of two independent measurements from freshly prepared samples.

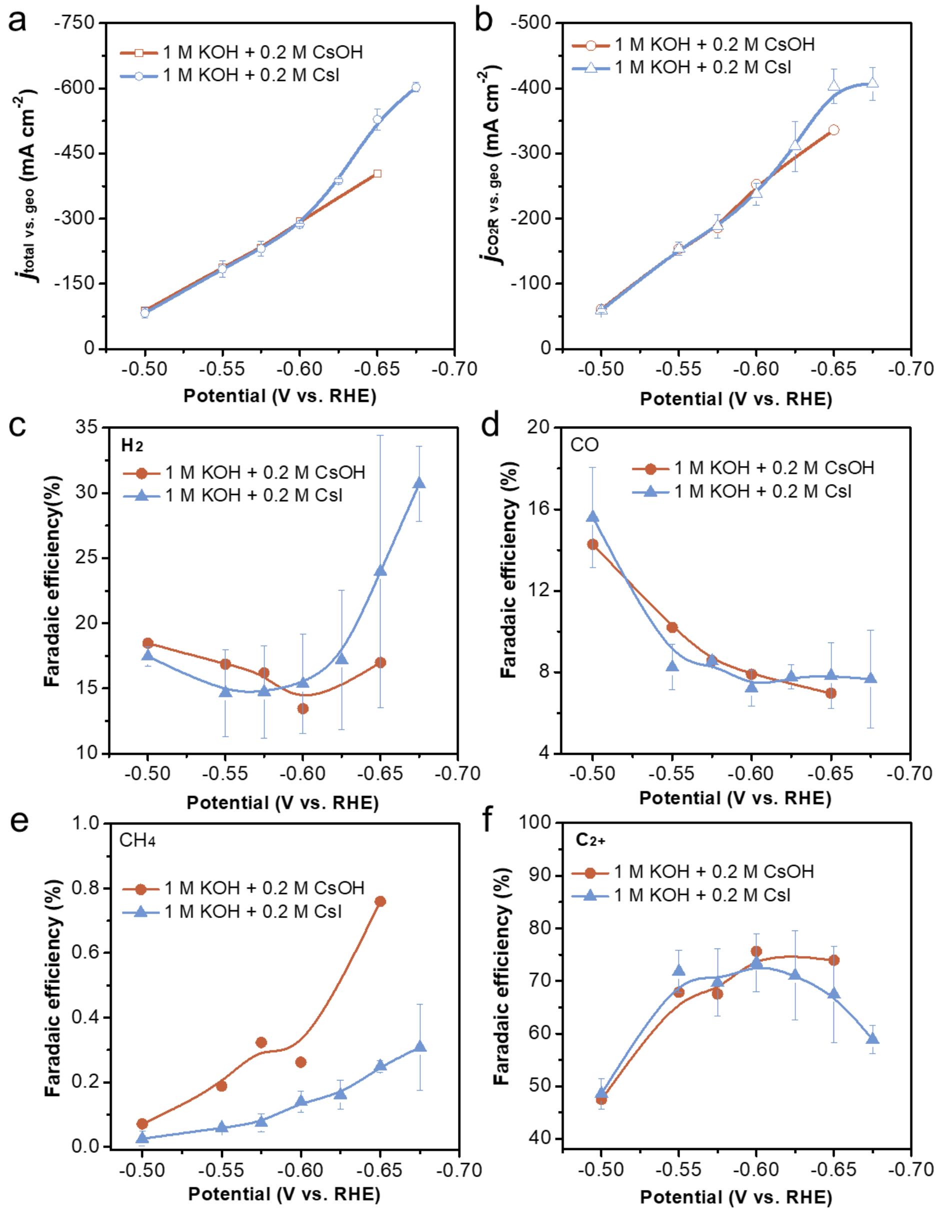

Supplementary Figure 15. Electrochemical performance in I^--containing and I^--free electrolytes. (a) Total current density, (b) partial current density of CO_2 reduction on CuNCs catalysts in CsOH-containing KOH electrolyte and CsI-containing KOH electrolyte; Faradaic efficiency of (c) H_2, (d) CO, (e) CH_4 and (f) C_{2+} products detected on CuNCs catalysts during CO_2 reduction in different electrolytes. The current density is normalized against geometric surface area. Each data point measured in CsI-containing KOH corresponds to the average of three independent chronopotentiometric measurements obtained from freshly-prepared samples and the error bar represents the standard deviation of these measurements. Each data point measured in CsOH-containing KOH electrolyte corresponds to the average value of two independent measurements from freshly prepared samples.

Additional data related to stability tests

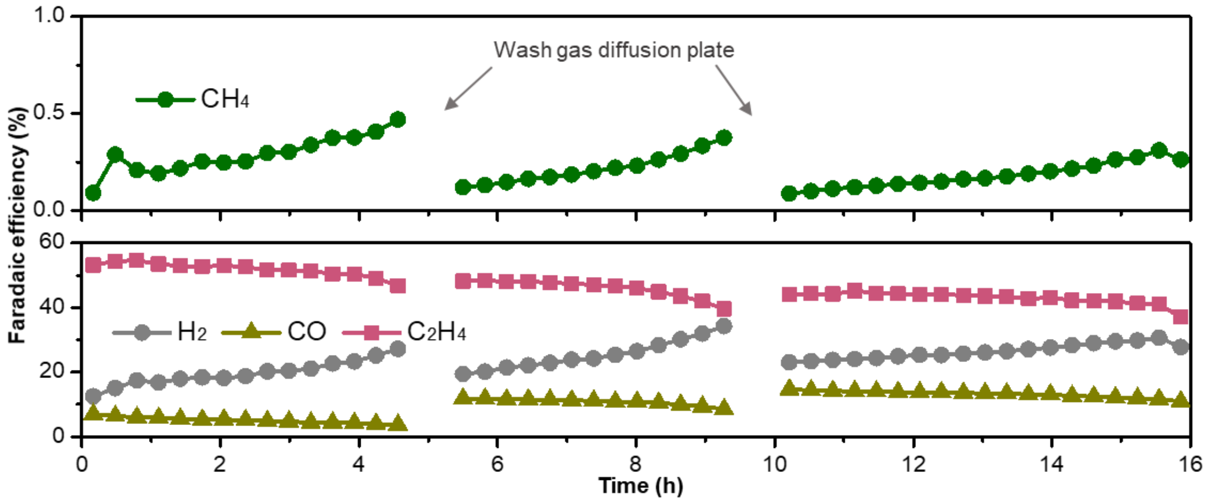

Supplementary Figure 16. Stability test of the CuNCs catalyst. Faradaic efficiency of CH_4, H_2, CO and C_2H_4 over 16 h-CO_2 reduction at -0.60 V vs. RHE (-273.7 mA cm^{-2}) on CuNCs catalyst.

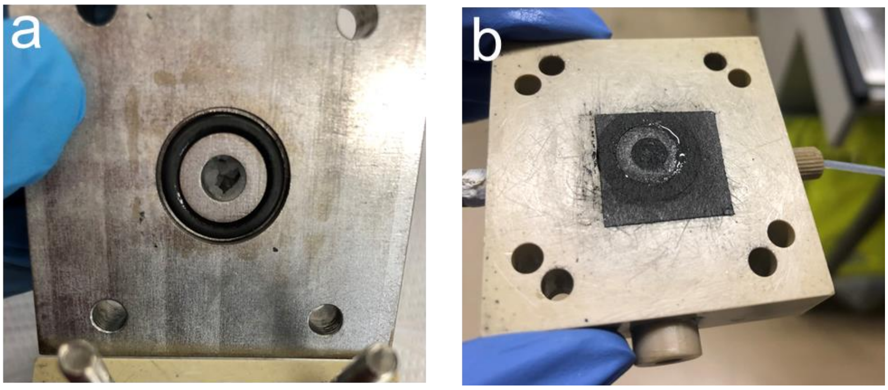

Supplementary Figure 17. Photographs of the chamber during stability test. Representative photographs of (a) the cathodic gas-flow plate and (b) the back of GDL after 4.5 h CO_2 reduction. Salt precipitates appeared inside the cathodic gas chamber as well as electrolyte droplets on the surface of carbon fiber were carefully washed out periodically to deconvolute their effects on the catalyst stability.

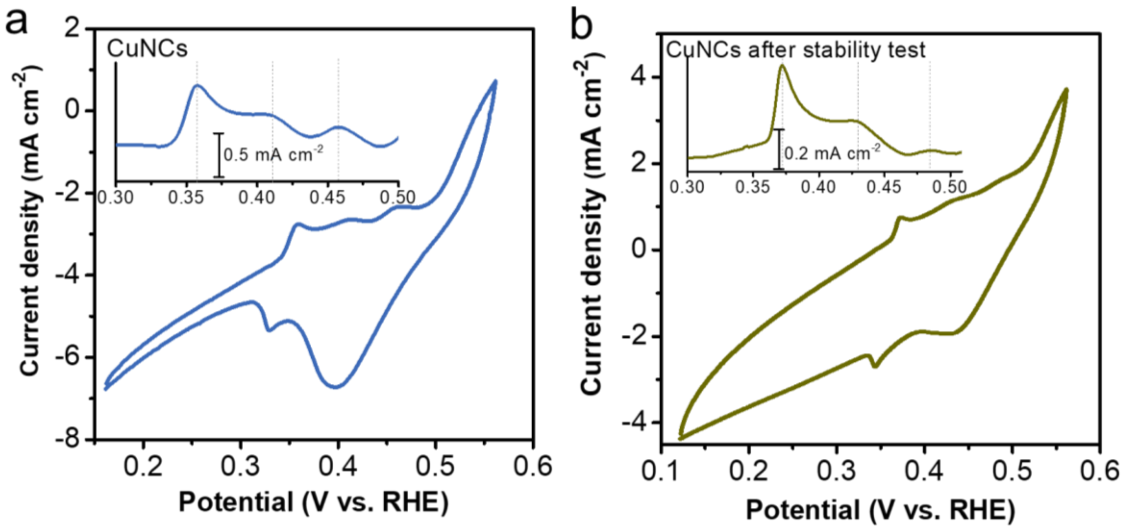

Supplementary Figure 18. OH^- adsorption measurements of as-prepared catalyst and catalyst after stability test. Cyclic voltammograms recorded on (a) as-synthesized CuNCs and (b) CuNCs after stabillity test at -0.60 V vs. RHE (-273.7 mA cm^{-2}) for ~16 h. The scan rates for curves in (a) and (b) are 50 and 30 mV s^{-1}, respectively. The inserted figures in (a) and (b) are the zoomed-in view of the anodic peaks of the cyclic voltammograms, showing OH^- adsorption peaks on Cu(100), Cu(110) and Cu(111) facets.

Supplementary data for identifying the key intermediates for propylene formation

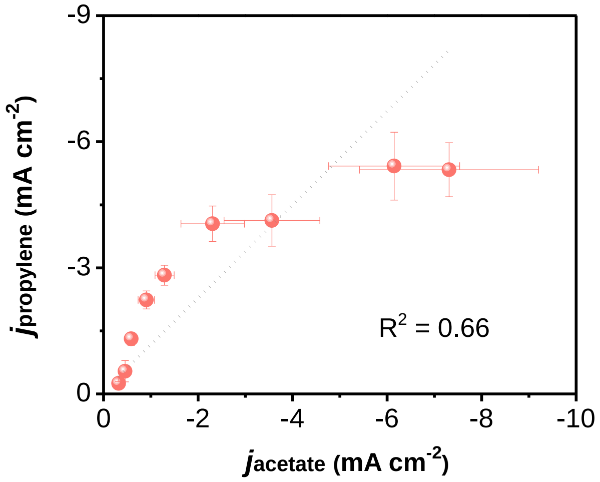

Supplementary Figure 19. Partial current density of propylene as a function of the one of acetate. The linearity between $j_{propylene}$ and $j_{acetate}$, as indicated by R^2 values of the fitting curves, is relatively poorer as compared to the one between propylene and ethylene/ethanol (Figure 3a-b).

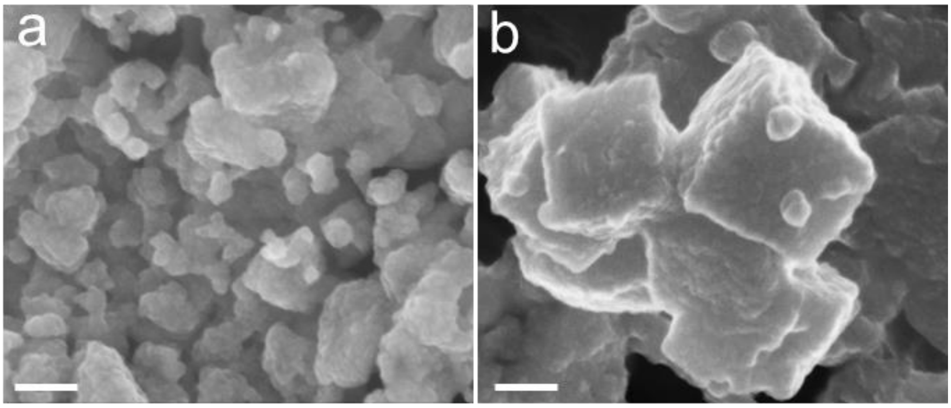

Supplementary Figure 20. Scanning electron micrographs of the catalysts under CO reduction condition. SEM images of (a) CuCl derived Cu after pre-reduction at -30 mA cm^{-2} for ~80 s and (b) CuNCs after CO reduction at -0.60 V vs. RHE for 10 min. Scale bars: 100 nm for a and b.

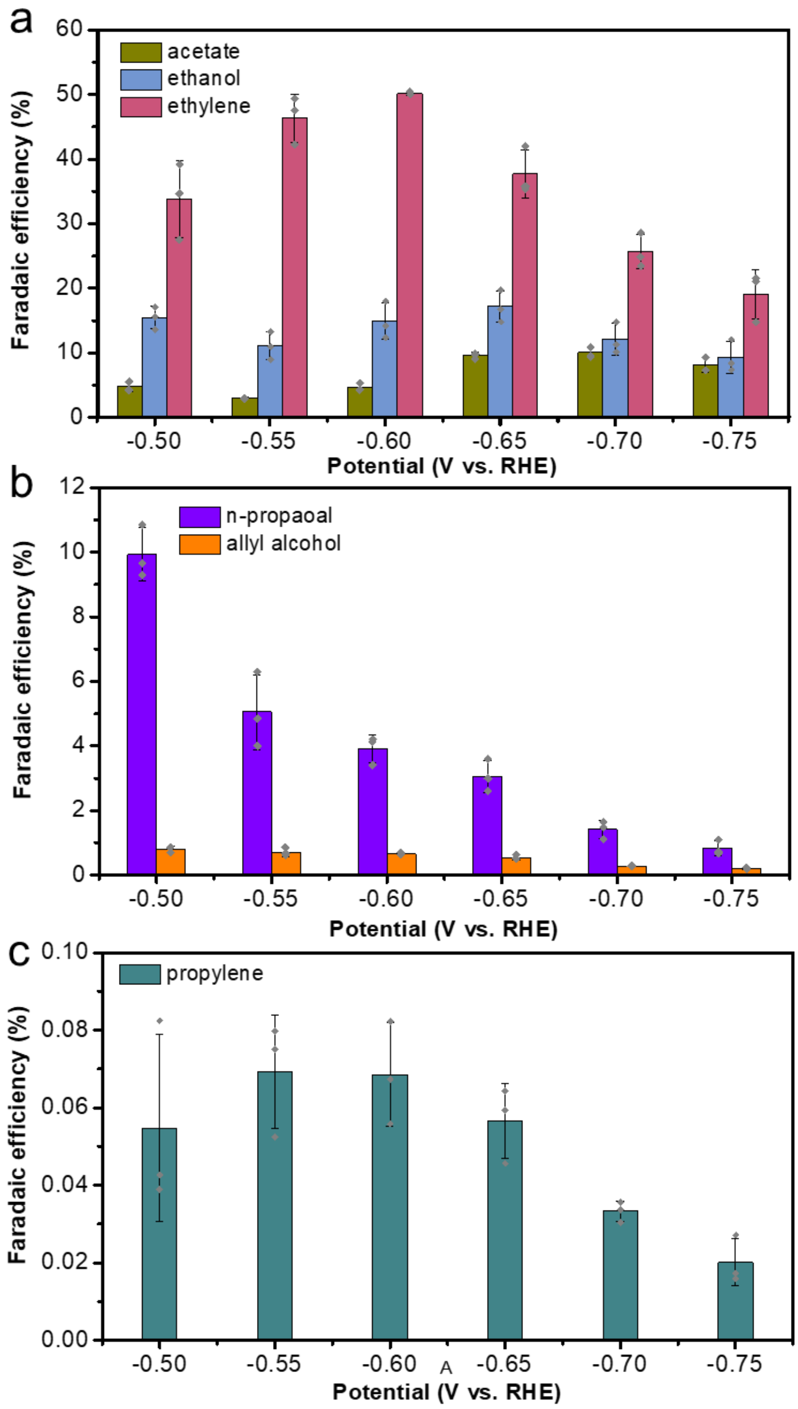

Supplementary Figure 21. Product distribution during CO reduction. Average Faradaic efficiency of (a) C_2 products including acetate, ethanol and ethylene and (b) C_3 products including *n*-propanol, allyl alcohol and (c) propylene produced on CuNCs catalysts during pure CO reduction at different potentials. Each data point corresponds to the average of three chronopotentiometric measurements obtained from freshly-prepared samples and the error bar represents the standard deviation of these measurements.

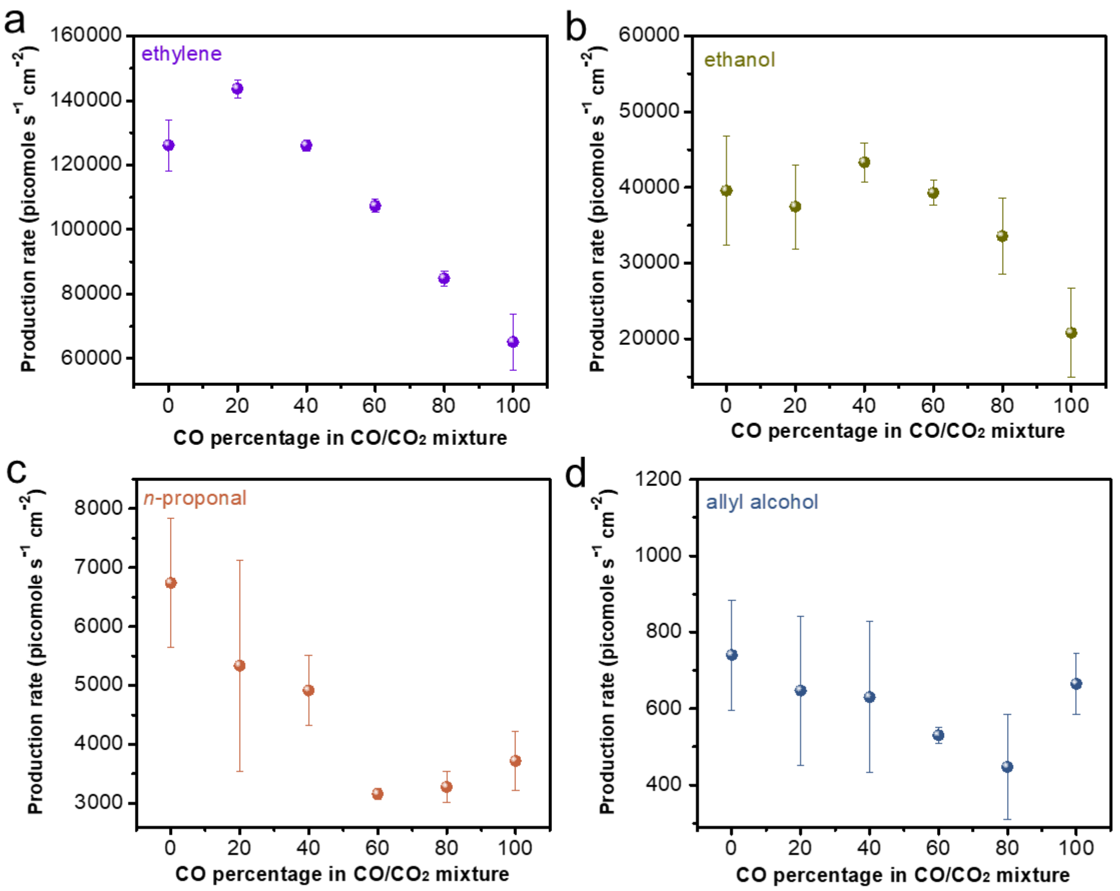

Supplementary Figure 22. Production rate of different products during CO_2-CO co-feeding experiments. Average absolute production rate of (a) ethylene, (b) ethanol, (c) *n*-propanol and (d) allyl alcohol is plotted as a function of different CO percentage in the mixture. Each data point corresponds to the average of three chronopotentiometric measurements obtained from freshly-prepared samples and the error bar represents the standard deviation of these measurements.

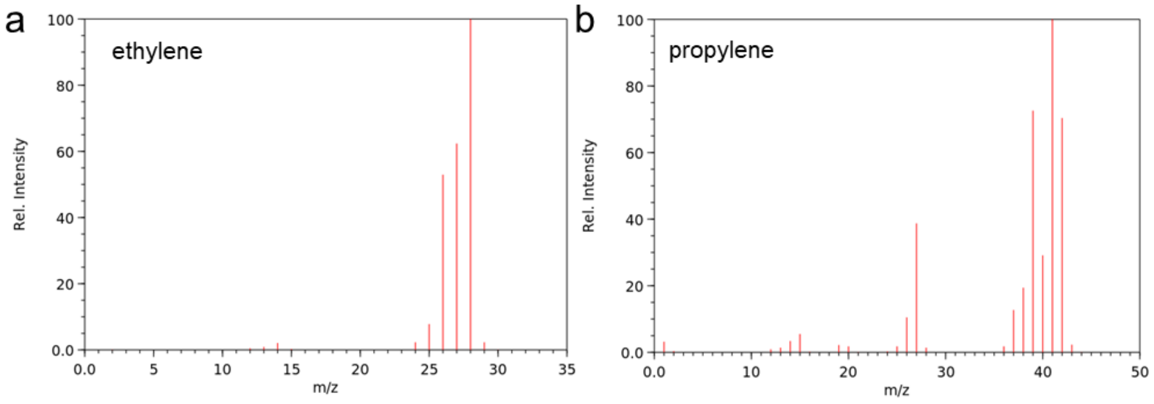

Supplementary Figure 23. Standard mass spectra of (a) ethylene and (b) propylene cited from NIST chemistry Webbook (https://webbook.nist.gov/chemistry).

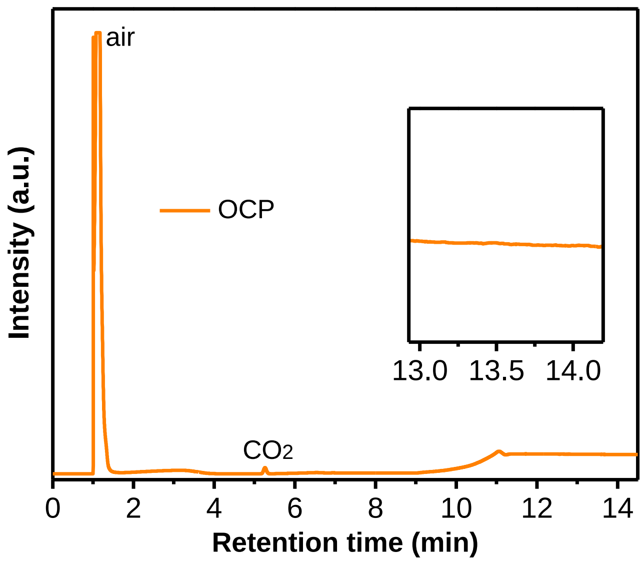

Supplementary Figure 24. Representative online gas chromatograph detected on CuNCs catalyst in 1 M KOH electrolyte containing allyl alcohol under open circuit potential.

Extended experiments for improved propylene formation

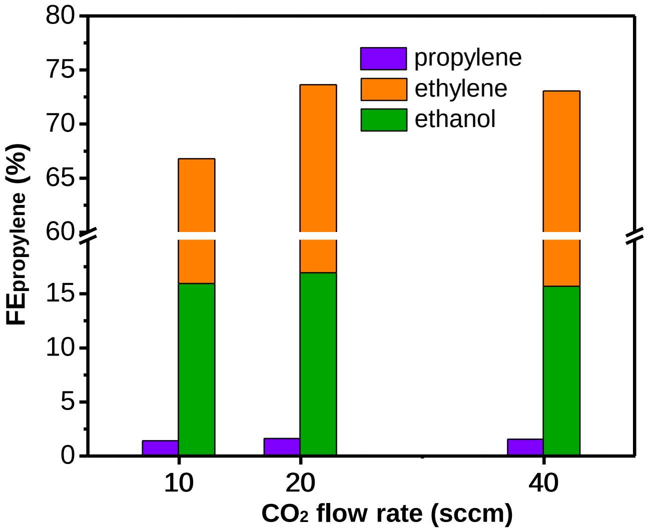

Supplementary Figure 25. Faradaic efficiency of propylene, ethylene and ethanol on CuNCs catalyst during CO_2 reduction with different gaseous flow rates. All the reduction reactions were performed at -0.60 V vs. RHE.

2. Supplementary Tables

Supplementary Table 1. Average cathodic total current density and faradaic efficiency of all the detected products from CO_2 reduction on CuNCs catalysts at different potential.

Potential (V vs. RHE)	Current density (mA cm^{-2})	Faradaic efficiency (%)											
		H_2	CO	CH_4	C_2H_4	C_2H_6	C_3H_6	$HCOO^-$	CH_3COO^-	C_2H_5OH	C_3H_7OH	C_3H_6O	Total
-0.475	60.71	20.13	15.91	0.00	24.94	0.06	0.42	8.48	0.57	6.57	3.52	0.36	80.96
-0.500	82.92	17.46	15.61	0.03	31.34	0.04	0.61	8.12	0.58	10.44	4.86	0.69	89.78
-0.525	131.58	16.48	10.73	0.04	41.99	0.04	1.02	5.14	0.41	10.54	3.68	0.39	90.46
-0.550	184.18	14.66	8.24	0.06	51.91	0.03	1.28	3.56	0.47	13.58	4.11	0.42	98.32
-0.575	231.25	14.74	8.54	0.07	48.48	0.03	1.26	3.15	0.53	15.01	3.95	0.46	96.22
-0.600	286.31	15.36	7.21	0.14	50.85	0.02	1.42	2.19	0.68	15.95	4.12	0.42	98.37
-0.625	387.78	17.20	7.76	0.16	50.48	0.02	1.09	1.19	0.83	15.61	2.73	0.28	97.36
-0.650	528.48	23.99	7.83	0.25	46.60	0.02	1.03	0.86	1.09	16.03	2.40	0.31	100.40
-0.675	602.53	30.69	7.66	0.31	38.40	0.01	0.89	0.71	1.17	15.75	2.34	0.33	98.25

Supplementary Table 2. Average cathodic partial current density of all the detected products from CO_2 reduction on CuNCs catalysts at different potential.

Potential	Partial current density (mA cm^{-2})										
(V vs. RHE)	H_2	CO	CH_4	C_2H_4	C_2H_6	C_3H_6	$HCOO^-$	CH_3COO^-	C_2H_5OH	C_3H_7OH	C_3H_6O
-0.475	12.23	9.66	0.00	15.14	0.03	0.25	5.15	0.35	3.99	2.14	0.22
-0.500	14.52	12.79	0.02	26.11	0.04	0.52	6.52	0.47	8.50	3.94	0.55
-0.525	21.76	14.12	0.06	54.94	0.05	1.34	6.65	0.53	13.70	4.75	0.52
-0.550	26.61	15.14	0.11	95.25	0.06	2.35	6.63	0.87	25.03	7.57	0.78
-0.575	34.36	19.73	0.18	111.96	0.06	2.91	7.24	1.23	34.83	9.05	1.04
-0.600	43.88	20.67	0.40	145.63	0.07	4.06	6.27	1.95	45.67	11.80	1.21
-0.625	66.55	30.12	0.62	195.94	0.07	4.23	4.62	3.22	60.57	10.60	1.09
-0.650	128.36	41.62	1.32	245.10	0.09	5.46	4.53	5.71	84.62	12.70	1.62
-0.675	184.96	45.99	1.85	231.33	0.07	5.35	4.26	7.02	94.84	14.12	1.97

Supplementary Table 3. Average cathodic total current density and faradaic efficiency of all the detected products from CO_2 reduction on poly-Cu catalysts at different potential.

Potential (V vs. RHE)	Current density (mA cm^{-2})	Faradaic efficiency (%)											
		H_2	CO	CH_4	C_2H_4	C_2H_6	C_3H_6	$HCOO^-$	CH_3COO^-	C_2H_5OH	C_3H_7OH	C_3H_6O	Total
-0.50	48.11	13.46	19.53	0.04	24.62	0.02	0.33	9.09	0.66	6.47	3.91	0.00	78.13
-0.525	70.12	15.57	19.56	0.14	31.26	0.00	0.43	7.83	0.67	10.14	4.05	0.48	90.13
-0.55	89.83	17.68	23.69	0.17	38.27	0.03	0.59	5.34	0.52	9.01	4.07	0.70	100.07
-0.575	119.11	15.22	16.15	0.39	49.37	0.03	0.86	3.68	0.51	11.08	3.52	0.72	101.53
-0.60	182.65	15.43	14.37	0.28	49.29	0.03	0.95	2.52	0.53	9.86	3.06	0.57	96.89
-0.625	246.89	13.77	8.67	0.29	52.62	0.02	1.11	2.00	0.64	15.46	4.07	0.49	99.14
-0.65	306.38	19.77	9.38	0.41	50.43	0.02	0.87	1.11	0.92	12.53	2.42	0.29	98.15
-0.675	411.36	28.07	9.33	0.80	43.25	0.01	0.70	0.84	1.18	12.02	1.95	0.25	98.40
-0.70	527.88	34.93	9.59	0.63	39.63	0.01	0.43	0.60	1.15	11.61	1.82	0.33	100.73

Supplementary Table 4. Average cathodic partial current density of all the detected products from pure CO_2 reduction on poly-Cu catalysts at different potential.

Potential (V vs. RHE)	Partial current density (mA cm^{-2})										
	H_2	CO	CH_4	C_2H_4	C_2H_6	C_3H_6	$HCOO^-$	CH_3COO^-	C_2H_5OH	C_3H_7OH	C_3H_6O
-0.500	6.48	9.40	0.02	11.84	0.01	0.16	4.37	0.32	3.11	1.88	0.00
-0.525	10.92	13.72	0.10	21.92	0.00	0.30	5.49	0.47	7.11	2.84	0.34
-0.550	15.88	21.28	0.15	34.38	0.03	0.53	4.80	0.47	8.10	3.66	0.63
-0.575	18.13	19.24	0.46	58.80	0.04	1.02	4.39	0.61	13.20	4.19	0.86
-0.600	28.18	26.25	0.52	90.02	0.05	1.74	4.61	0.96	18.01	5.59	1.05
-0.625	33.99	21.40	0.73	129.91	0.06	2.73	4.93	1.59	38.16	10.05	1.21
-0.650	60.57	28.75	1.24	154.51	0.06	2.67	3.40	2.83	38.39	7.41	0.88
-0.675	115.46	38.37	3.31	177.90	0.06	2.89	3.46	4.86	49.43	8.04	1.03
-0.700	184.40	50.65	3.30	209.19	0.05	2.25	3.16	6.08	61.31	9.62	1.73

Supplementary Table 5. The charge values for Pb UPD on different surface facets of CuNCs and poly-Cu catalysts. The values were estimated from the recorded cyclic voltammograms shown in Supplementary Figure 8 and 11. The reference charges of Pb UPD on Cu(100) and Cu(111) single crystals are 262 and 285 μC cm^{-2}, respectively.

Catalyst	Charge consumed for deposition on Cu(100) (mC cm^{-2})	Charge consumed for deposition on Cu(111) (mC cm^{-2})	Surface area of facet Cu(100) (cm^2)	Surface area of facet Cu(111) (cm^2)	Cu(111)/Cu(100)	Roughness factor
CuNCs/GDL	5.15 [a]	7.35	6.48	8.51	1.31	45.42
Poly-Cu/GDL	1.86	5.49	2.34	6.36	2.72	26.36
CuNCs/FTO	1.26 [b]	1.09	3.15	2.51	0.80	8.63
Poly-Cu/FTO	0.64 [c]	1.47.	2.51	5.31	2.12	7.60

[a] The surface area of both CuNCs and poly-Cu measured in GDL based flow cell is 0.33 cm^2.

[b] The surface area of CuNCs/FTO is 0.656 cm^2.

[c] The surface area of poly-Cu/FTO is 1.029 cm^2.

Supplementary Table 6. The charge values for OH^- adsorption on different facets of CuNCs and poly-Cu catalysts. The values were estimated from the recorded cyclic voltammograms shown in Supplementary Figures 9 and 10. The reference charges of OH^- adsorption on Cu(100) and Cu(111) single crystals are 8.22 and 2.16 μC cm^{-2}, respectively.

Catalyst	Charge consumed for OH^- adsorption on Cu(100) (mC cm^{-2})	Charge consumed for OH^- adsorption on Cu(111) (mC cm^{-2})	Surface area of Cu(100) (cm^{-2})	Surface area of Cu(111) (cm^{-2})	Cu(111)/Cu(100)
As-syn CuNCs	0.172 [a]	0.060	6.91	9.23	1.34
after 10 min reaction	0.173	0.065	6.95	9.85	1.42
As-syn Poly-Cu	0.051	0.053	2.05	8.06	3.93
after 10 min reaction	0.052	0.052	2.09	8.01	3.83

[a] The surface area of both CuNCs and poly-Cu measured in GDL based flow cell is 0.33 cm^2.

Supplementary Table 7. The charge values for OH^- adsorption on different facets of as-synthesized CuNCs catalyst and the one after stabillity test at -273.7 mA cm^{-2} for ~16 h. The values were estimated from the cyclic voltammograms shown in Supplementary Figure 23. The reference charges of OH^- adsorption on Cu(100) and Cu(111) single crystals are 8.22 and 2.16 μC cm^{-2}, respectively.

Catalyst	Charge of Cu(100) (mC cm^{-2})	Charge of Cu(111) (mC cm^{-2})	Surface area of Cu(100) (cm^{-2})	Surface area of Cu(111) (cm^{-2})	Cu(111)/Cu(100)
As-synthesized CuNCs	0.220	0.0726	8.82	11.09	1.26
CuNCs after 16 h-reaction	0.168	0.0463	6.76	7.08	1.05

Supplementary Table 8. Absolute production rate of the detected products during mixed CO_2-CO reduction at -0.60 V vs. RHE with different CO_2 and CO flow-rate ratio.

CO_2:CO flow-rate ratio	Current density ($mA\ cm^{-2}$)	absolute production rate ($picomole\ s^{-1}\ cm^{-2}$)								
		H_2	CH_4	C_2H_4	C_3H_6	$HCOO^-$	CH_3COO^-	C_2H_5OH	C_3H_7OH	C_3H_6O
10:0	286.31	232276.34	557.80	126138.95	2351.99	30772.85	2391.04	39583.58	6739.39	740.66
8:2	281.45	256876.87	3259.27	144857.72	2941.15	20976.07	3106.96	37458.69	5335.35	647.49
6:4	271.65	363857.22	3692.75	125928.45	2035.27	14874.21	6151.37	43324.05	4914.14	630.30
4:6	257.44	534348.25	5456.60	108434.09	1440.54	6310.72	8966.40	39295.54	3159.09	530.22
2:8	189.61	403032.09	5914.28	85309.25	1211.58	3505.96	13970.90	33565.34	3278.43	447.33
0:10	100.06	113118.33	5900.11	63037.91	91.35	N.D.[a]	12594.66	20791.88	3720.71	665.18

[a] N.D.: not detected

Supplementary Table 9. Average cathodic total current density and faradaic efficiency of all the products generated from CO reduction on CuNCs catalysts at different potential.

Potential (V vs. RHE)	Current density (mA cm^{-2})	Faradaic efficiency (%)									
		H_2	CH_4	C_2H_4	C_2H_6	C_3H_6	CH_3COO^-	C_2H_5OH	C_3H_7OH	C_3H_6O	Total
-0.50	39.26	15.22	8.01	33.76	0.06	0.05	4.77	15.46	9.93	0.80	88.06
-0.55	61.55	16.46	5.28	46.34	0.04	0.07	2.96	11.12	5.05	0.71	88.03
-0.60	100.06	20.72	3.54	50.13	0.03	0.07	4.72	14.87	3.91	0.66	98.65
-0.65	171.42	28.80	2.20	37.68	0.01	0.06	9.61	17.15	3.06	0.55	99.12
-0.70	304.44	40.48	1.75	25.64	0.01	0.03	10.05	12.10	1.42	0.28	91.76
-0.75	450.76	47.11	1.27	19.11	0.01	0.02	8.09	9.32	0.83	0.20	85.95

Supplementary Table 10. Average cathodic partial current density of all the products generated from CO reduction on CuNCs catalysts at different potential.

Potential (V vs. RHE)	Partial current density (mA cm^{-2})								
	H_2	CH_4	C_2H_4	C_2H_6	C_3H_6	CH_3COO^-	C_2H_5OH	C_3H_7OH	C_3H_6O
-0.50	5.78	3.03	12.68	0.02	0.02	1.80	5.92	3.80	0.30
-0.55	9.91	3.18	27.85	0.02	0.04	1.78	6.70	3.04	0.43
-0.60	21.51	3.64	51.69	0.03	0.07	4.89	15.37	4.02	0.68
-0.65	48.81	3.72	63.77	0.02	0.10	16.30	29.05	5.18	0.93
-0.70	122.64	5.30	77.75	0.04	0.10	30.47	36.69	4.30	0.84
-0.75	212.64	5.75	86.31	0.03	0.09	36.52	42.07	3.76	0.88

Supplementary Table 11. The propylene production rate and allyl alcohol (AA) feeding rate during the electroreduction of allyl alcohol that dissolved in 1 M KOH with different concentrations.

AA feeding conc. (mM)	AA residual concentration (mmol)	produced propylene concentration (picomole)	AA feeding rate (mmol s^{-1} cm^{-2})	propylene production rate (mmol s^{-1} cm^{-2})
1	0.999	7.427	1.263×10^{-5}	1.875×10^{-7}
5	4.068	178.196	6.313×10^{-5}	1.800×10^{-6}
10	9.311	110.764	12.626×10^{-5}	2.797×10^{-6}
25	22.115	811.580	31.566×10^{-5}	8.198×10^{-6}
50	46.060	646.474	63.131×10^{-5}	1.633×10^{-5}

Supplementary Table 12. Calculated conversion efficiency during CO_2 reduction on the CuNCs at different potentials. Each data point shown here corresponds to the average of three chronopotentiometric measurements from freshly-prepared samples.

Potential (V vs. RHE)	AA. feeding (production) rate mmol s^{-1} cm^{-2}	partial propylene reaction rate (mmol s^{-1} cm^{-2})	total propylene reaction rate (mmol s^{-1} cm^{-2})	conversion efficiency
-0.475 V	4.037×10^{-7}	1.050×10^{-8}	4.360×10^{-7}	2.41%
-0.500 V	1.009×10^{-6}	2.623×10^{-8}	8.911×10^{-7}	2.94%
-0.525 V	9.082×10^{-7}	2.361×10^{-8}	2.263×10^{-7}	1.04%
-0.550V	1.413×10^{-6}	3.673×10^{-8}	3.862×10^{-6}	0.95%
-0.575V	1.917×10^{-6}	4.985×10^{-8}	4.757×10^{-6}	1.05%
-0.600V	2.220×10^{-6}	5.772×10^{-8}	6.147×10^{-6}	0.94%
-0.625V	1.917×10^{-6}	4.985×10^{-8}	7.100×10^{-6}	0.70%
-0.650V	2.624×10^{-6}	6.821×10^{-8}	9.362×10^{-6}	0.73%
-0.675 V	3.734×10^{-6}	9.708×10^{-8}	7.766×10^{-6}	1.25%

3. Supplementary Reference

1 Popovic, S., Bele, M. & Hodnik, N. Reconstruction of Copper Nanoparticles at Electrochemical CO2 Reduction Reaction Conditions Occurs via Two-step Dissolution/Redeposition Mechanism. *ChemElectroChem* **8**, 2634-2639 (2021).

2 Raaijman, S. J., Arulmozhi, N. & Koper, M. T. M. Morphological Stability of Copper Surfaces under Reducing Conditions. *ACS Appl. Mater. Interfaces* **13**, 48730-48744 (2021).
