# Supplementary figures and images for: Electrochemical synthesis of propylene from carbon dioxide on copper nanocrystals
Source: Nat Chem. 2023 Apr 6;15(5):705–13. doi: 10.1038/s41557-023-01163-8 (PMC10159857; doi:10.1038/s41557-023-01163-8)

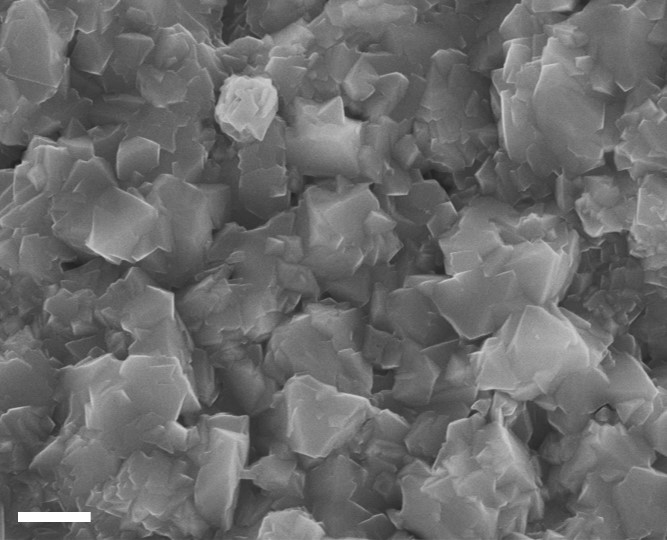

Supplement: Source Data Fig. 1 — Source data for XRD and XPS, and unprocessed electron micrographs. [file 41557_2023_1163_MOESM2_ESM.zip › Source Data Fig.1/Source Data Fig. 1c.jpg]

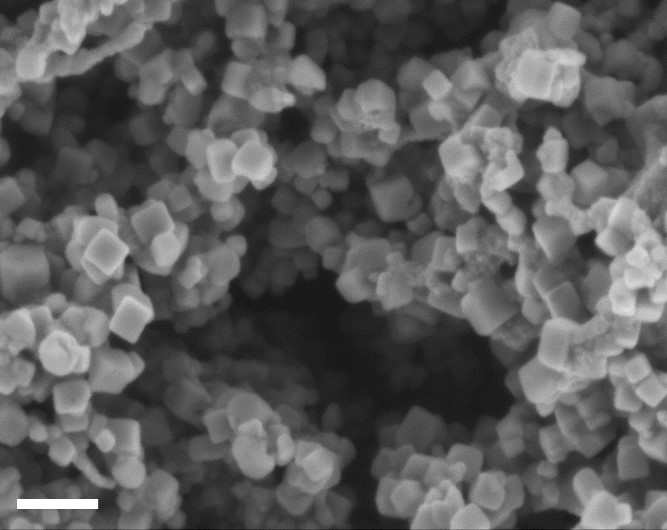

Supplement: Source Data Fig. 1 — Source data for XRD and XPS, and unprocessed electron micrographs. [file 41557_2023_1163_MOESM2_ESM.zip › Source Data Fig.1/Source Data Fig. 1d.jpg]

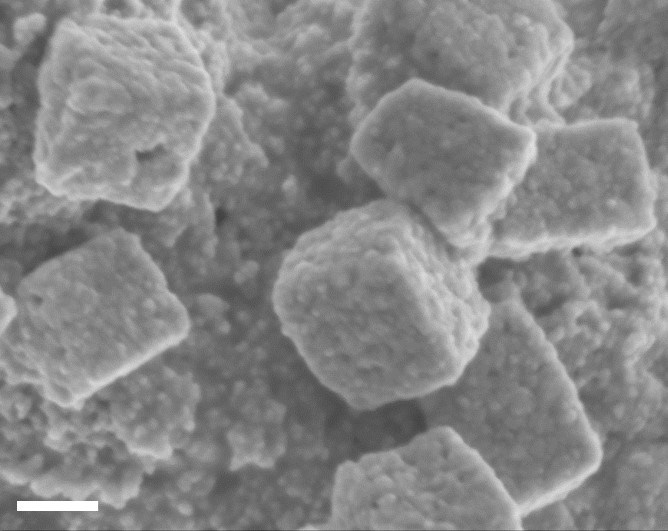

Supplement: Source Data Fig. 1 — Source data for XRD and XPS, and unprocessed electron micrographs. [file 41557_2023_1163_MOESM2_ESM.zip › Source Data Fig.1/Source Data Fig. 1e.jpg]

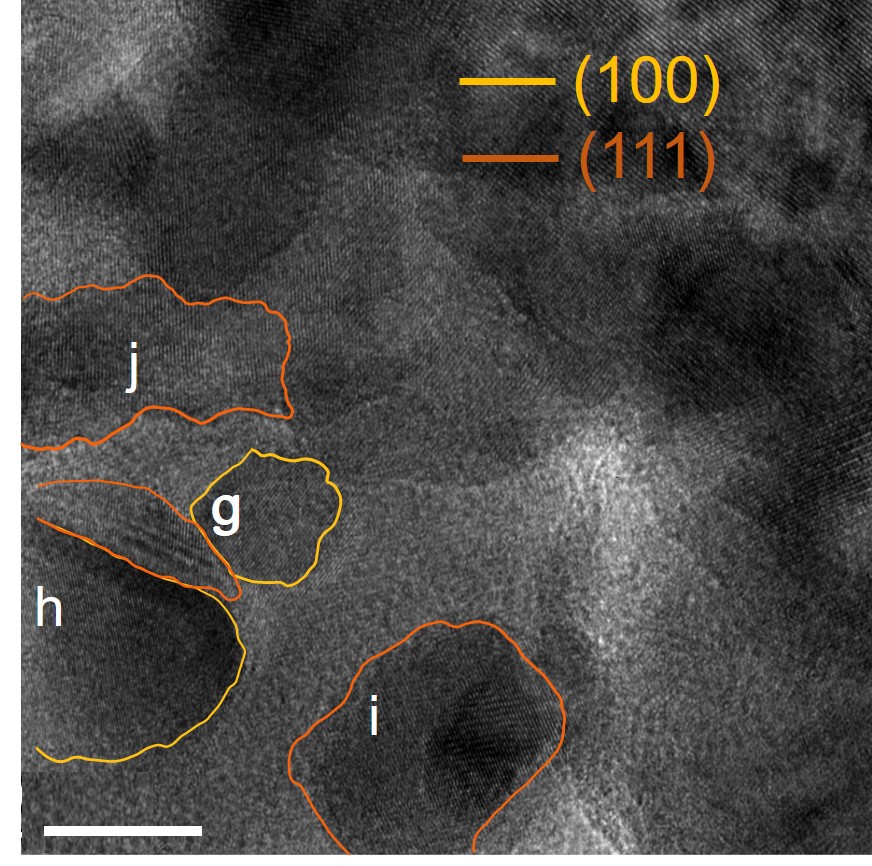

Supplement: Source Data Fig. 1 — Source data for XRD and XPS, and unprocessed electron micrographs. [file 41557_2023_1163_MOESM2_ESM.zip › Source Data Fig.1/Source Data Fig. 1f.jpg]

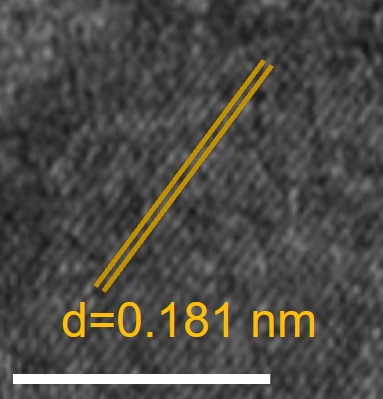

Supplement: Source Data Fig. 1 — Source data for XRD and XPS, and unprocessed electron micrographs. [file 41557_2023_1163_MOESM2_ESM.zip › Source Data Fig.1/Source Data Fig. 1g.jpg]

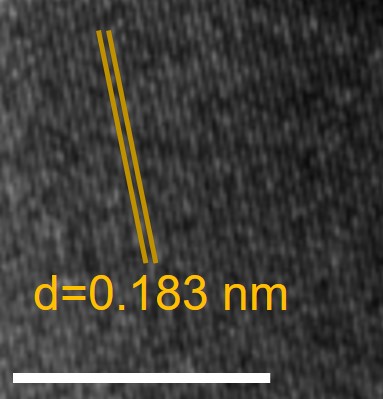

Supplement: Source Data Fig. 1 — Source data for XRD and XPS, and unprocessed electron micrographs. [file 41557_2023_1163_MOESM2_ESM.zip › Source Data Fig.1/Source Data Fig. 1h.jpg]

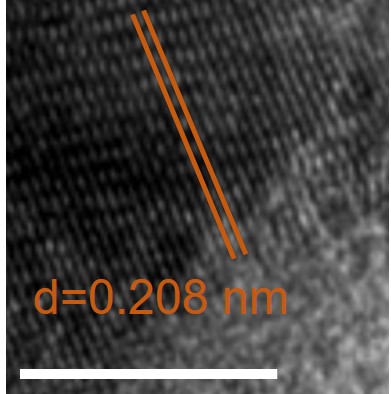

Supplement: Source Data Fig. 1 — Source data for XRD and XPS, and unprocessed electron micrographs. [file 41557_2023_1163_MOESM2_ESM.zip › Source Data Fig.1/Source Data Fig. 1i.jpg]

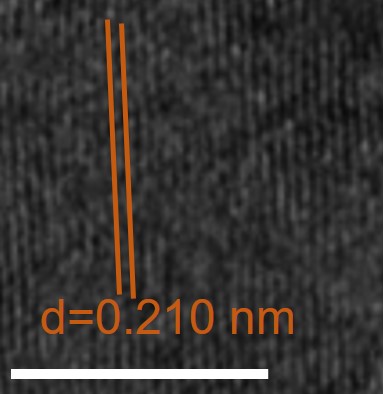

Supplement: Source Data Fig. 1 — Source data for XRD and XPS, and unprocessed electron micrographs. [file 41557_2023_1163_MOESM2_ESM.zip › Source Data Fig.1/Source Data Fig. 1j.jpg]

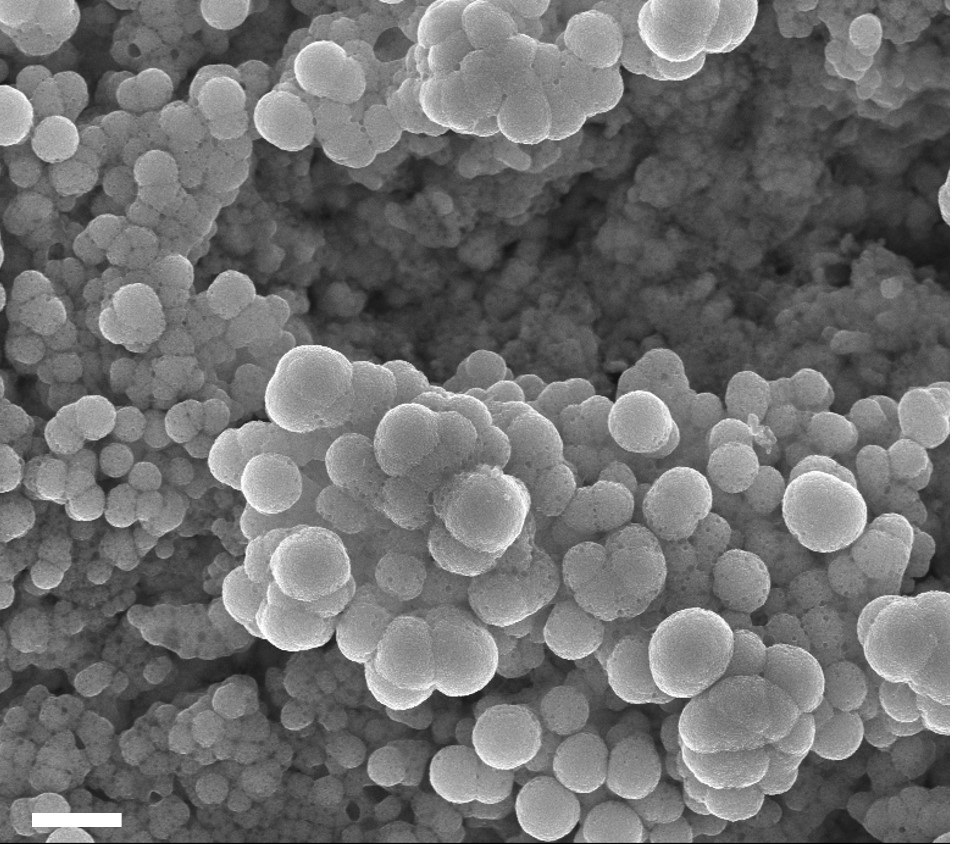

Supplement: Source Data Extended Data Fig. 5 — Unprocessed electron micrographs [file 41557_2023_1163_MOESM10_ESM.zip › Gao_SourceData_ED_Fig. 5a.jpg]

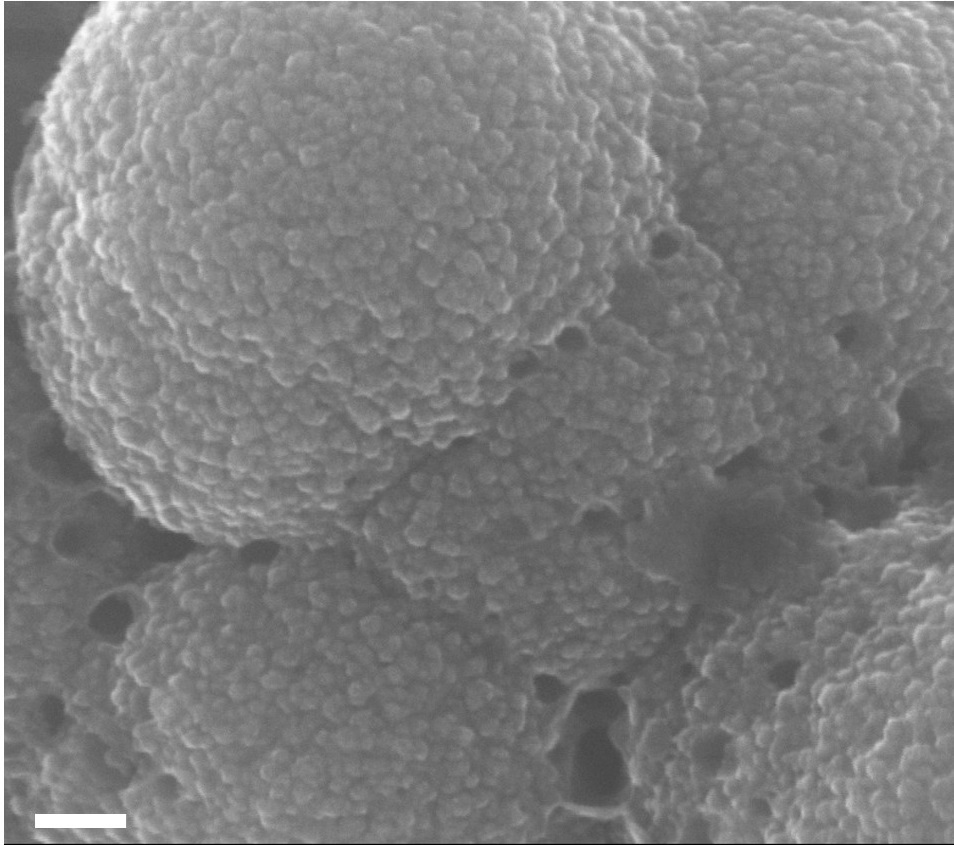

Supplement: Source Data Extended Data Fig. 5 — Unprocessed electron micrographs [file 41557_2023_1163_MOESM10_ESM.zip › Gao_SourceData_ED_Fig. 5b.jpg]
